# Improved Therapeutic Efficiency of Senescent Cell‐specific, Galactose‐Functionalized Micelle Nanocarriers

**DOI:** 10.1002/smll.202405732

**Published:** 2024-12-18

**Authors:** Badri Parshad, Andrew George Baker, Ishtiaq Ahmed, Alejandra Estepa‐Fernández, Daniel Muñoz‐Espín, Ljiljana Fruk

**Affiliations:** ^1^ Department of Chemical Engineering and Biotechnology University of Cambridge Philippa Fawcett Drive Cambridge CB3 0AS UK; ^2^ Early Cancer institute Department of Oncology University of Cambridge Hills Road Cambridge CB2 0XZ UK

**Keywords:** amphiphiles, drug delivery, galactose, navitoclax, self‐assembly, senescent cells

## Abstract

Cellular senescence has recently been recognized as one of the hallmarks of cancer, aging, as well as many age‐related disorders, sparking significant interest in the development of senolytics, compounds that can remove senescent cells. However, most current pharmacological strategies face challenges related to non‐specific delivery, leading to significant side effects that hinder safe and effective treatments. To address these issues, galactose‐functionalized amphiphiles are synthesized that can self‐assemble into micelles and be loaded with a senolytic cargo. These galactose‐micelles are responsive to the lysosomal β‐galactosidase enzyme, present in elevated amounts in senescent cells, and are employed for specific delivery of the senolytic Bcl2‐inhibitor Navitoclax. This novel formulation showed reduced delivery and toxicity to non‐senescent cells, thereby increasing the senolytic index of Navitoclax and making it suitable for future in vivo experimental designs to improve selectivity and safety profiles.

## Graphical abstract

Farewell, Therapy‐induced Senescence: Galactose‐coated micelles enable selective delivery of senolytic drug into senescent cells while sparing healthy ones. This enzyme‐responsive system enhances specificity to senescent cells arising after chemotherapy, representing a significant advancement in improving post‐chemotherapy health.

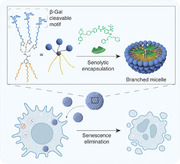

## 1 Introduction

Senescence is a cellular response to damage or stress whereby cells enter a state of permanent cell cycle arrest and secrete pro‐inflammatory cytokines and chemokines.^[^ ^1^, ^2^ ^]^ This senescence‐associated secretory phenotype (SASP) plays numerous roles within the tumor microenvironment contributing to inflammation and tumorigenesis.^[^ ^1^, ^3^ ^]^ Cellular senescence commonly inhibits the proliferation of damaged cells and triggers tissue repair, which protects organisms from cancer or damage.^[^ ^4^ ^]^ However, there is mounting evidence that accumulation of senescent cells caused by persistent damage, for example, accumulated during ageing, also plays an antagonistic role which can result in inflammation and further tissue damage ultimately leading to multiple age‐related disorders such as cancer, Alzheimer's, fibrosis, cardiovascular diseases, and diabetes.^[^ ^1^, ^5^, ^6^ ^]^ Chemotherapy‐induced senescence has also been linked to cancer recurrence and metastasis, and there have been increased efforts to develop strategies for removal of these cells as a part of growing efforts to improve post‐chemotherapy health.^[^ ^4^, ^7^, ^8^ ^]^

The clearance of these cells in mouse models has been shown to improve both lifespan and healthspan,^[^ ^6^, ^9^, ^10^ ^]^ increasing interest in developing senotherapies, therapeutic strategies aimed at the clearance of senescent cells.^[^ ^11^, ^12^ ^]^ Currently, numerous laboratories in the academia and more than 20 companies worldwide are working on different senescence‐related therapies and number of them are in different stages of clinical trials.^[^ ^13^ ^]^ However, despite these efforts, no senolytic was yet approved, and targeting senescent cells to increase the efficacy and reduce the side effects of existing strategies is still a major challenge.^[^ ^7^, ^14^ ^]^

The increasing efforts to improve drug bioavailability and achieve target specificity have led to significant advancements in controlled drug delivery using nanosized carriers for senescent cells.^[^ ^8^, ^15^ ^]^ Most of developed drug nanocarriers and pro‐drug systems have relied on exploitation of β‐galactosidase (SA‐β‐gal) enzyme activity in the lysosomes of senescent cells in a colorimetric reaction.^[^ ^8^, ^16^ ^]^ SA‐β‐gal cleaves glycosidic bond of a bound galactose sugar, and the addition of galactose to the senolytic small molecule drug Navitoclax has been shown to significantly lower toxic side effects in chemotherapy‐induced senescence.^[^ ^7^, ^8^ ^]^ In addition, the coating of porous silica nanoparticles with galactose units provided both improved delivery and specific release of the drug cargo post‐galactose cleavage in presence of the enzyme.^[^ ^8^, ^17^, ^18^ ^]^ Although very promising, porous silica is not an ideal nanocarrier due to its potential toxicity, liver accumulation, and general issues with intravenous application of inorganic nanoparticles.^[^ ^19^, ^20^ ^]^

Despite a number of studies demonstrating galactose modifications to improve targeting drugs to senescent cells, such synthesis procedures modify the parent drug structure and also have lengthy synthesis procedures.^[^ ^7^, ^21^, ^22^ ^]^ Inspired, by earlier studies by Munoz‐Espin et al who demonstrated that the encapsulation of Navitoclax, a potent Bcl2 family of antiapoptotic proteins inhibitor, within porous silica nanocarriers improves drug safety profiles and efficacy in models of chemotherapy‐ and damage‐induced senescence,^[^ ^8^ ^]^ we sought the use of micelles to improve the safety profiles. To minimize any adverse effects stemming from the nanocarrier itself, amphiphilic micelle system was designed and explored for delivery of the senolytic Navitoclax.

Micelles have already been used in clinic to deliver small molecules such as chemotherapeutic epirubicin for improved breast cancer treatment (NC‐6300, NCT03168061) and paclitaxel (Genexol‐PM, NCT02739633) for solid tumors, with both systems being characterized by high loading capacity and well tolerated.^[^ ^23^, ^24^, ^25^ ^]^ In addition to excellent drug loading properties, self‐assembly of amphiphilic micelles can be tuned by modifying the structure of building blocks, which results in a range of possible morphologies that can impact the cell uptake and delivery.^[^ ^26^ ^]^

Three distinct types of amphiphiles, linear, twinned, and branched, were prepared and investigated for the delivery of a model drug, Nile red, and the senolytic Navitoclax. Among these, branched structures demonstrated the best safety profile and drug loading capacity. The organic biocompatible structure, small size, enzyme‐cleavable, and adaptability to different drug cargo, makes micelles a versatile platform for drug delivery. Moreover, small molecular drugs can be encapsulated and delivered specifically to the target cells without additional chemical modification of the cargo. To the best of our knowledge, this is the first report to describe and compare different galactose‐functionalized amphiphiles in terms of self‐assembly, drug encapsulation, and in vitro senolytic behavior. Besides their excellent drug loading properties, the use of click chemistry for micelle modification provides a modular approach, allowing various functionalities to be added to the surface of the nanocarriers. This versatility extends their potential applications beyond senescent cell therapy.

## 2 Results and Discussion

### 2.1 Synthesis of Amphiphilic Building Blocks

**Scheme 1:**
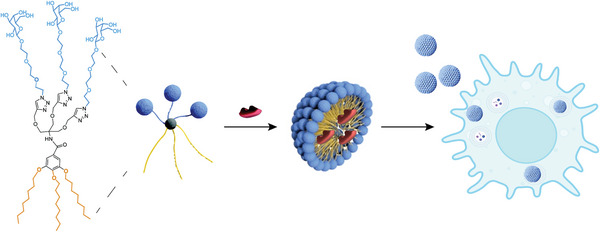
Amphiphilic building blocks containing galactose sugar are assembled into micelles that can deliver drug cargo (red) into senescent cells.

Biocompatible, galactose‐functionalized, low molecular weight amphiphiles were prepared which can form stable micellar structures in water. These micelles contain inward facing hydrophobic alkyl chains and the hydrophilic, galactose‐containing chains on their surface. The hydrophobic core serves as a reservoir for hydrophobic drugs, while the hydrophilic shell ensures stability in biological systems (**Scheme** **1**).

**Figure 1:**
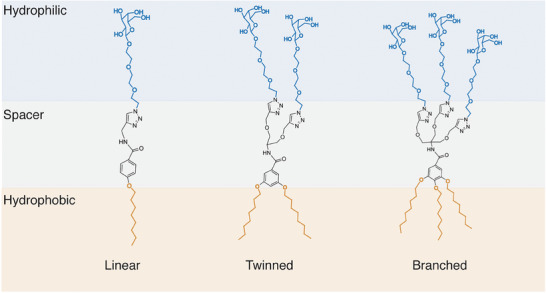
Structure of linear twinned and branched amphiphiles with their three distinct regions (hydrophobic and hydrophilic chains separated by a spacer moiety) employed for micelle formation.

Three distinct amphiphiles; linear, twinned, and branched characterized by a different degree of functionalization but similar hydrophobic‐hydrophilic balance were first prepared (**Figure** **1**) to be used for the assembly of micelles.

First, amino moieties (Figure S1, Supporting Information. **4b**/**4c**) for the amide coupling to construct the hydrophobic units were prepared employing slightly modified reported methods I, Figure S1a, Supporting Information). The amino group of commercially available serinol (**1b**) and tris‐ or tris(hydroxymethyl)aminomethane (**1c**) was protected by using di‐*tert*‐butyl dicarbonate in ethanol to afford Boc‐protected amino compounds (**2b**/**2c**). The Boc‐protected serinol and tris HCl were treated with propargyl bromide and sodium hydroxide to afford terminal alkynes (**3b**/**3c**). The deprotection of Boc groups using trifluoroacetic acid afforded amino compounds (**4b**/**4c**), which was confirmed by the disappearance of the protons and carbon signals of Boc group in ^1^H NMR and ^13^C NMR. To enhance the solubility of the amphiphiles in water and provide the cleavage side for to β‐S‐gal, the glycosyl azide (**7**) containing acetyl protected β‐galactose was synthesized according to the literature procedure (I, Figure S1b, Supporting Information). 2‐[2‐(2‐Chloroethoxy)ethoxy]ethanol (**5**) was treated with sodium azide in DMF to give mono‐azide (**6**) which reacted with pentaacetyl galactose in the presence of BF_3_.Et_2_O in dry DCM afforded glycosyl azide (**7**) confirmed by the appearance of acetate and sugar protons peaks in NMR (Figures S2 and S3, Supporting Information).

Once the precursor molecules were obtained, the systematic synthesis of the amphiphiles was carried out by coupling of alkyl chain(s) containing aromatic hydrophobic moieties with galactosylated hydrophilic moieties using Cu‐mediated click chemistry (Figure S4, Supporting Information).

**Scheme 2:**
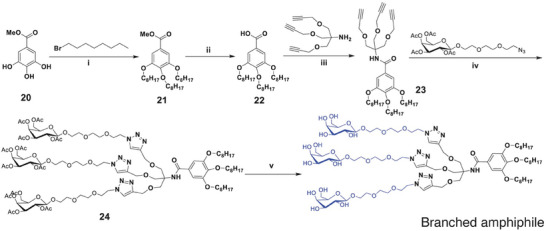
Synthesis of the branched hydrophobic moiety and subsequent Cu‐mediated click coupling to afford amphiphilic branched building block. i) K_2_CO_3_, acetonitrile, 50 °C, 16 h; ii) 2N aq. NaOH, THF, rt, 5 h; iii) EDC.HCl, HOBt, DIPEA, DMF, rt, 16 h; iv) CuSO_4_.5H_2_O, sodium ascorbate, THF, H_2_O, 50 °C, 16 h; v) 2N aq. NaOH, THF, rt, 5 h.

Hydrophobic moieties were prepared by a multi‐step approach resulting in the presence of octyl chain(s) linked to aromatic ring through ether linkage. Aromatic functionality was introduced to enhance the stability of supramolecular assemblies and improve interactions between building blocks through *π–π* stacking. Commercially available core units including methyl‐4‐hydroxybenzoate (**8**) for linear amphiphile, methyl 3,5‐dihydroxybenzoate (**14**) for twinned amphiphile, and methyl 3,4,5‐trihydroxybenzoate (**20**) for branched amphiphile were used for the synthesis of hydrophobic tails (**Scheme** **2**; ESI, Figure S4, Supporting Information).

The hydrophobic moieties (**10**, **16**, and **22**) were developed employing these cores (Figure S4, Supporting Information). The hydrophobic units **9**, **15**, and **21** bearing ester linkage were synthesized by *O*‐alkylation of the aromatic hydroxy groups in core units using 1‐bromooctane in the presence of potassium carbonate as a base and acetonitrile as a solvent to create the methyl ester hydrophobic core, Figures S5–S10, Supporting Information). The de‐esterification of compounds (**9**, **15**, and **21**) was performed using sodium hydroxide in THF resulting in acid derivatives (**10**, **16**, and **22**) (Figures S11–S16, Supporting Information).

The amide coupling of the aromatic acids and acetylene amines (**4a–c**) was performed in DMF using *N*‐(3‐dimethylaminopropyl)‐*N*‐ethyl‐carbodiimide hydrochloride (EDC HCl) and *N*‐hydroxybenzotrizole (HOBt) to afford hydrophobic components (**11**, **17**, and **23**) with terminal alkynes (Figures S17–S22, Supporting Information). The acetylene was coupled to hydrophilic moiety employing CuSO_4_.5H_2_O and sodium ascorbate in THF/H_2_O. The successful click reaction resulted in formation of protected amphiphiles (**12**, **18**, and **24**) and was confirmed by the disappearance of the terminal acetylenic proton signal at δ 2.5 ppm (, Figures S23–S28, Supporting Information). A comparison of the integrated area for the one, two, and three terminal methyl groups of the hydrophobic chains (δ 0.90–0.99 ppm in the ^1^H NMR spectra) with the multiplet/singlet ≈δ 8.0 ppm for the triazolyl ring protons confirmed the formation of one, two, and three triazolyl rings respectively. Additional proof was obtained by ^13^C NMR spectra and the appearance of triazolyl ring carbons (C‐5 and C‐4/C‐5″ and C‐4″) ≈δ 124–142 ppm. In addition, triazole ring formation resulted in a characteristic shift from δ 4.1 ppm to ≈δ 4.5 ppm in the ^1^H NMR spectra for the methylene protons of the hydrophobic moieties placed in the vicinity of the amide bond of triazolyl rings (Figures S23–S28, Supporting Information).

The final step in the synthesis of amphiphilic building blocks was a base‐catalysed deprotection, which afforded desired linear, twinned, and branched amphiphiles as confirmed by NMR (Figures S29–S34, Supporting Information). HR‐MS data indicated the presence of [M+Na]^+^ and/or [M+2Na]^2+^ peaks, which are in accordance with their molecular formula: [M+H]^+^ was 625.34 for linear amphiphile C_30_H_49_N_4_O_10,_ [M+H]^+^ 1202.6654 m z^−1^ for twinned C_56_H_95_N_7_O_21_, and 1757.9422 m z^−1^ for branched C_80_H_138_N_10_O_31_Na.

### 2.2 Micelle Assembly and Characterisation

**Figure 2:**
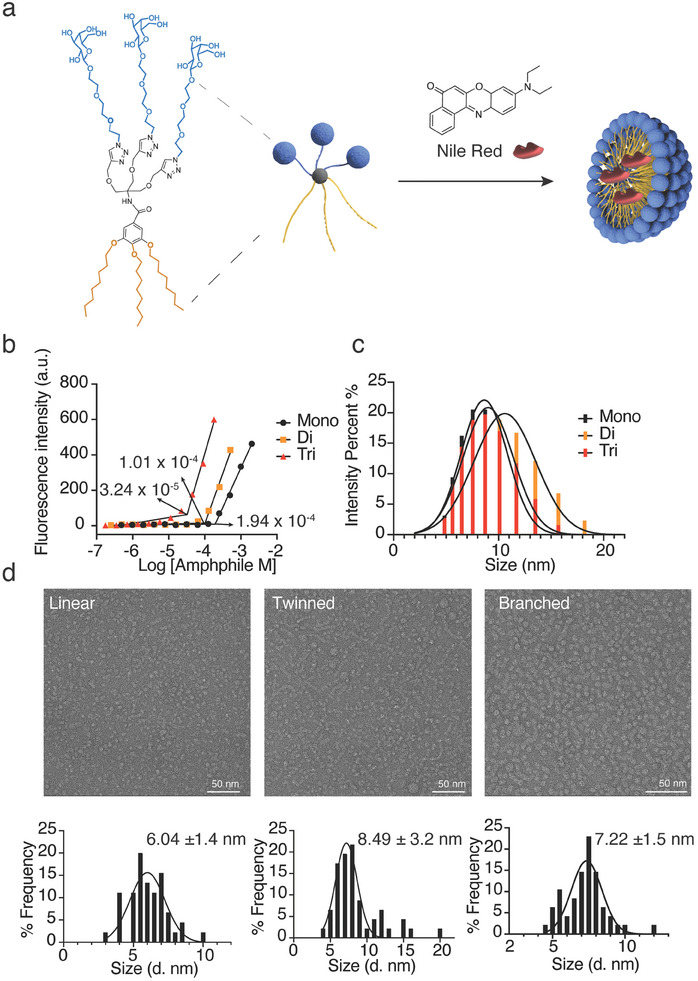
Characterization of micelles prepared using galactose‐modified amphiphiles. a) Scheme of micelle formulation and Nile Red loading. b) Fluorescence intensity of Nile Red versus concentration of amphiphile to determine CMC of the micelles. Measurements are done in aqueous solution at 25 °C. c) DLS intensity distribution profile of amphiphiles (1.0 mg mL^−1^ in PBS pH 7.4). d) TEM micrographs of linear, twinned, and branched micelles with spherical structures obtained for linear (d = 6.04 ± 1.4 nm) and branched (d = 7.22 ± 1.5 nm), and worm‐like and spherical structures obtained for twinned amphiphile (d = 8.49 ± 3.2 nm). d denotes diameter.

Once the synthesis of building blocks was completed, micelles were prepared by stirring amphiphiles overnight at room temperature in PBS. To assess the differences between three classes of prepared amphiphiles, the critical micelle concentration (CMC), the minimum concentration at which the amphiphilic systems start forming micelles, was determined. Nile red methods were utilized to obtain CMC values whereby the fluorescence increase of Nile red encapsulated in a non‐polar environment was monitored (**Figure** **2**a). A fixed amount of Nile red and varied the amount of amphiphile over a range of concentrations (10^−7^ to 10^−3^ m) was employed in the study resulting in CMC values of 1.94 × 10^−4^ m for linear, 1.01 × 10^−4^ m for twinned and 3.24 × 10^−5^ m for branched amphiphiles (Figure 2b). Interestingly, CMC for branched amphiphiles was significantly lower than for the linear or twinned structures although all amphiphiles have similar hydrophilic‐lipophilic balance (HLB, Table S1, Supporting Information). This can be attributed to a significantly larger content of hydrophilic and hydrophobic building blocks in branched structures, which facilitates the aggregation at lower concentration.^[^ ^27^ ^]^

The assembly of the amphiphiles was also studied by dynamic light scattering (DLS) at a concentration of 1.0 mg mL^−1^ (Figure 2c) resulting in homogenous size distribution with values of 8.64 ± 2.2, 10.59 ± 2.9, and 9.01 ± 2.4 nm for linear, twinned and branched structure respectively. A slightly larger average size was observed for twinned systems, and this is most likely due to the presence of additional nanostructures as confirmed by TEM. Slight increase observed in twinned and branched structures compared to linear is in line with earlier studies reporting the correlation between the overall area of amphiphilic building blocks and the size of obtained micelles.^[^ ^28^ ^]^

To assess the intrinsic morphology of prepared amphiphiles, TEM measurements were performed for all three amphiphiles at a concentration of 1.0 mg mL^−1^ (Figure 2d; Figure S35, Supporting Information). While spherical structures are observed for assembly of liner and branched amphiphiles (6.04 ± 1.4 and 7.22 ± 1.5 nm, respectively), both spherical and wormlike structures were observed for twinned amphiphiles (8.49 ± 3.2 nm).

### 2.3 β‐Galactosidase‐Responsive Release of Nile Red In Vitro

**Figure 3:**
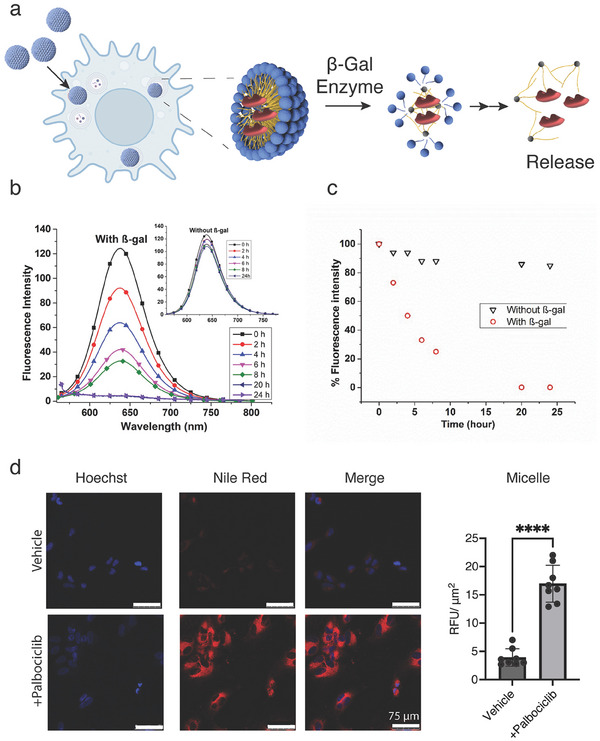
Encapsulation and release studies of Nile Red, and in vitro delivery. a) Scheme of Nile red release in senescent cells; b) Change in fluorescence intensity of encapsulated Nile red in branched amphiphile in the presence and absence (inset) of β‐galactosidase at 37 °C over the period of 24 h; c) Percentage release of Nile red calculated from fluorescence intensity. d) Confocal images of SK‐MEL‐103 cells incubated with demdriitic micelle loaded with Nile red. The nucleus was stained using Hoechst stain and confocal images were obtained on a Leica SP5 confocal microscope. Data is obtained using confocal images, and represent mean ± SD, and a Two tailed t test was used to calculate the significance (^*^ *p* <0.05, ^**^ *p* <0.01, ^***^ *p* <0.001, and ^****^ *p* < 0.0001).

Owing to its limited solubility in aqueous medium, Nile red is often used to investigate the encapsulation efficiency (**Figure** **3**a) and cellular uptake of micellar nanostructures and other nanocarriers,^[^ ^29^ ^]^ and was used to explore the loading of prepared galactose‐modified micelles. The addition of 0.12 mg of Nile red to 5.0 mg mL^−1^ of amphiphile solution in water avoided any cargo aggregation and resulted in maximum encapsulation. To quantify the encapsulated Nile red, lyophilization of the encapsulated amphiphiles was carried out followed by dissolution in methanol and subsequent recording of their absorbance spectra. The encapsulation efficiency was calculated by using molar extinction coefficient of Nile Red (45000 M^−1^ cm^−1^ at 552 nm, Figure S36, Supporting Information) and Table S2, Supporting Information) with highest loading observed for branched amphiphile (2.78% mol/mol) followed by twinned (1.10% mol/mol) and linear (0.94% mol/mol) micelles. In addition to quantification, we assessed the morphological changes in the micelles after encapsulation. The twinned micelles transitioned to a more homogeneous spherical form, as previously reported.^[^ ^30^ ^]^ In contrast, the branched micelles retained their original morphology, with no significant size difference between loaded and unloaded spherical micelles (Figure S37, Supporting Information). Based on these results, we chose to focus further studies on branched micelles due to their high cargo‐loading capacity and monodisperse spherical structure.

Following the successful encapsulation of Nile Red, β‐galactosidase mediated release was explored by exposing loaded amphiphiles to the solution of β‐galactosidase (lacA) enzyme from *Aspergillus oryzae*. As expected, a time‐dependent decrease of fluorescence intensity of Nile red loaded amphiphile is observed at 37 °C in the presence of β‐gal, indicating the release of Nile red from the hydrophobic core of the nanostructures as they disassemble upon cleavage of β‐galactose moiety (Figure 3b). In branched amphiphile, 50% of fluorescence intensity is lost within 6 h, with more than 90% of Nile red released after 24 h (Figure 3c). In the control experiment without the addition of β‐galactosidase enzyme, less than 6% decay in fluorescence intensity of Nile red occurs, suggesting that amphiphiles do not release the drug in the absence of β‐galactosidase.

Once β‐galactosidase mediated release was confirmed, micelle uptake and response were studied in senescent cells. Two models of chemotherapy‐induced senescence were employed, specifically Palbociclib‐induced senescence in melanoma cell line SK‐MEL‐103 and cisplatin‐induced senescence in lung cancer A549 cells. To evaluate the activation of the senescence program in these models, SA‐β‐gal (senescence‐associated β‐gal) activity was investigated using a commercially available staining kit, while enhanced expression of p21 cell cycle inhibitor and loss of phosphorylated retinoblastoma (Rb) protein were determined by Western blot (Figure S36, Supporting Information).

Prior to cell uptake studies, the toxicity of the unloaded branched micelles on both senescent and non‐senescent cells was explored indicating the absence of cytotoxicity (Figure S39, Supporting Information). Next, branched micelles were loaded with Nile red, and cellular uptake was evaluated. We found significantly (p < 0.0001) more uptake and delivery of Nile Red‐micelles in senescent SK‐MEL‐103 cells (Figure 3d) as well as A549 (p < 0.001, Figure S40, Supporting Information) than control non‐senescent cells. Further, the signal colocalizes with the lysosome of the senescent cells where the β‐galactosidase enzyme is present in high amounts (Figure S40, Supporting Information). Finally, we compared Nile Red (3.0 µm) on its own at an equivalent concentration to Nile red loaded micelles. While Nile Red on its own was 1.7x more selective for senescent SK‐MEL‐103 cells (p < 0.01), micelle formulation resulted in 3.5x (p < 0.0001) more cargo present in senescent cells compared to control non‐senescent cells (Figure S41, Supporting Information), indicating improved uptake ratios which can contribute to therapeutic efficiency of the formulated drug cargo.

### 2.4 In vitro Activity of Navitoclax‐Loaded Branched Amphiphiles

We then performed studies with a model senolytic drug Navitoclax. Here we encapsulated Navitoclax within a branched micelle with obtained loading of 4.45% (mol/mol). Navitoclax is a potent senolytic compound that targets the Bcl2 family of proteins and, as a result, the anti‐apoptotic (pro‐survival) pathways that are upregulated in senescent cells.^[^ ^31^ ^]^

**Figure 4:**
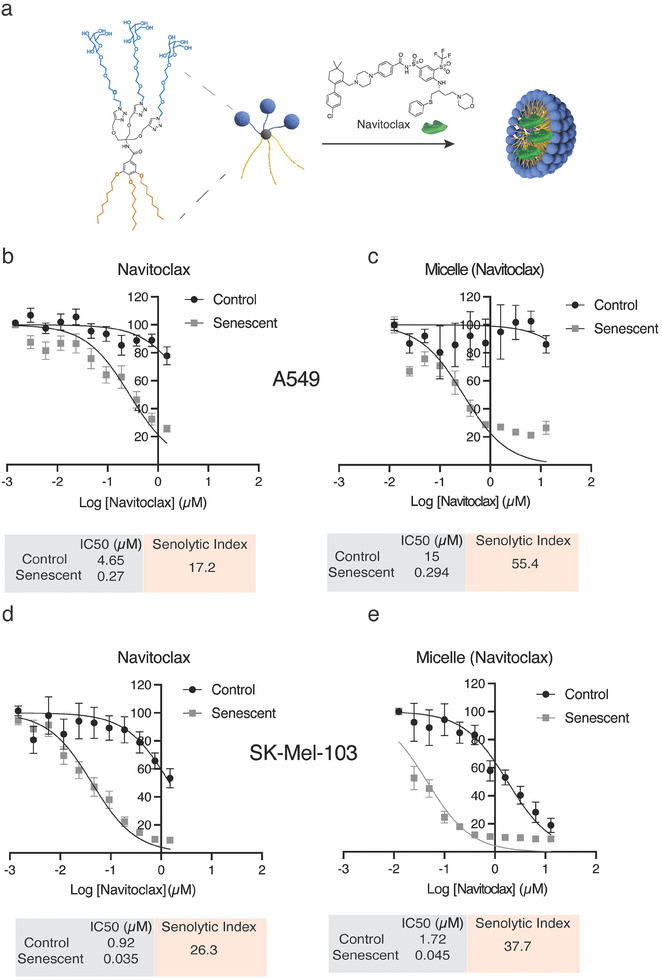
In vitro, study of Navitoclax‐loaded branched micelles. a) Scheme of Navitoclax loading. b) Quantification of IC50 values for Navitoclax and its dedritic micelle formulation using Cell Titer Blue viability assay on A549 (b,c) and SK‐MEL‐103 (d,e) cells. The senolytic index was calculated as the ratio of IC50 for control and senescent cells.

To determine if the micelle formulation enhances the delivery of the senolytic drug, we compared the toxicity of free Navitoclax versus micelle‐encapsulated Navitoclax (**Figure** **4**a) in lung cancer A459 and melanoma SK‐MEL‐103 cell lines. The concentration required to induce 50% of death (IC50) after 72 h of treatment was 4.65 µm for A459 control cells and 0.27 µm for cisplatin‐induced A459 senescent cells in case of free Navitoclax, while for micelle‐encapsulated Navitoclax values increase significantly for control cells (15.0 µm) while no significant increase was observed for cisplatin‐induced senescent cells (0.294 µm) (Figure 4b,c). These values were used to obtain the senolytic index, a measure of the efficacy of a senolytic drug,^[^ ^32^ ^]^ this is calculated as the ratio of the IC50 values of the control cells to the IC50 value of the senescent cells. For the A549 cells, this increased when navitoclax was encapsulated in the micelle, from 17.2 to 55.4 respectively. A similar trend was observed in Palbociclib‐induced SK‐MEL‐103 senescent cells (Figure 4d,e), with IC50 values of 0.92 and 1.72 µm for non‐senescent cells, and 0.035 and 0.045 µm for Palbociclib‐induced senescent cells for free and micelle‐encapsulated Navitoclax respectively. These values resulted in an increase of the senolytic index from free (26.6) to encapsulated (37.7) Navitoclax indicating an improved therapeutic effect of branched micelle formulations due to the protection of non‐senescent cells from the cytotoxic activity of the drug.

## 3 Conclusion

The successful synthesis of liner, twinned, and branched amphiphiles enabled the preparation of non‐toxic micelle nanocarriers, which can be loaded with a model drug Nile red and senolytic Navitoclax. Micelles made from the branched amphiphiles were shown to have the lowest critical micelle concentration maintaining its structure even in dilute conditions,^[^ ^33^ ^]^ as well as the largest loading capacity. Owing to the presence of hydrophilic galactose heads, and the marginal lysosomal β‐galactosidase activity in non‐senescent cell, micelle formulation resulted in lower amount of drug cargo delivered to non‐senescent cells compared to the free drug. Such selectivity can be exploited to selectively deliver potent senolytic drugs such as Navitoclax to senescent cells, displaying a high lysosomal SA‐β‐gal activity, while reducing the cargo toxicity in non‐senescent cells, ultimately resulting in a significantly improved senolytic index. Future work will focus on in vivo study of these nanostructures with particular emphasis on potential reduction of side effects typically associated with Navitoclax.

## 4 Experimental Section

All the solvents and chemicals used in the synthesis of amphiphiles were procured from Merck, VWR International and Fisher Chemicals. Nile red and Navitoclax used in the encapsulation studies were purchased from BioServ and APExBIO Technology LLC respectively. The progress of the chemical reactions was monitored using TLC plate (Merck silica gel 60 F_254_) with visualization of the spots on TLC using a UV lamp, cerric solution, and 5% sulfuric acid in ethanol. MilliQ Water was used for preparing the samples for physio‐chemical studies.

### NMR Method

^1^H and ^13^C NMR spectra of the amphiphiles were recorded on Jeol 400 MHz spectrometer and referencing was done by using residual peak from deuterated solvent. CDCl_3_, DMSO‐*d_6_*, and D_2_O were used to measure the spectra. The chemical shift value is shown on δ scale, and the coupling constant values are in Hertz and represented by *J*. The mass data in Supporting Information were recorded on Agilant mass spectrometers.

### Synthesis of Amphiphile Precursors

Detailed synthesis protocols for compounds 7–24 are given in ESI.

### Synthesis of Linear/Twinned/Branched Amphiphile

In a 100 mL round bottom, an aqueous solution of sodium hydroxide (1N aq. NaOH, 1 mL) was added to a solution of compound **12**/**18**/**24** (100 mg) in THF (2.5 mL). The resulting mixture was stirred at rt for 5 h. On completion of the reaction, the solution was neutralized with the addition of an ion exchange resin (Dowex 50WX8). The solvent was filtered and removed under reduced pressure followed by lyophilization to afford the desired **Linear**/**Twinned**/**Branched** amphiphile.

### Linear Amphiphile

Obtained as white solid in 84% yield; ^1^H NMR (400 MHz, D_2_O): δ = 7.81 (s, 1H), 7.67 (d, J = 7.0 Hz, 2H), 6.57 (d, J = 7.7 Hz, 2H), 4.45 (bs, 2H), 4.34–4.28 (m, 3H), 3.92–3.87 (m, 2H), 3.79–3.67 (m, 4H), 3.64–3.49 (m 7H), 3.43–3.20 (bs, 4H), 1.46 (br, 2H), 1.30–1.14 (m, 10H), 0.8 (t, J = 6.3 Hz, 3H); ^13^C NMR (100.5 MHz, CDCl_3_): δ = 167.41, 161.38, 144.77, 129.15, 125.42, 124.07, 113.73, 102.86, 75.05, 72.73, 70.71, 69.65, 69.43, 69.40, 68.62, 68.54, 68.40, 60.89, 49.72, 31.91, 29.58, 29.32, 29.15, 26.00, 22.58, 13.84 ppm; ESI: calculated for C_30_H_49_N_4_O_10_ [M+H]^+^ 625.3443, found 625.3419.

### Twinned Amphiphile

Obtained as white solid in 80% yield; ^1^H NMR (400 MHz, D_2_O): δ = 8.00 (s, 2H), 7.06 (bs, 2H), 6.43 (bs, 2H), 4.67–4.52 (m, 6H), 4.48–4.42 (m, 4H), 4.08–3.99 (m, 4H), 3.94–3.58 (m, 30H), 1.79–1.08 (m, 24H), 0.9 (bs, 6H); ^13^C NMR (100.5 MHz, CDCl_3_): δ = 181.32, 167.42, 159.89, 144.02, 135.79, 124.81, 102.92, 75.06, 72.77, 70.76, 69.74, 69.57, 69.50, 68.77, 68.56, 68.46, 63.53, 60.88, 49.86, 31.80, 29.30, 28.98, 26.01, 23.16, 22.69, 13.86 ppm; ESI: calculated for C_56_H_95_N_7_O_21_ [M+H]^+^ 1202.6654, found 1202.6682.

### Branched Amphiphile

Obtained as white solid in 78% yield; ^1^H NMR (400 MHz, D_2_O): δ = 7.94 (s, 3H), 7.08 (bs, 3H), 4.63‐4.51 (m, 10H), 4.40‐4.29 (m, 4H), 4.03–3.42 (m, 52H), 1.63–1.19 (m, 36H), 0.81 (bs, 9H); ^13^C NMR (100.5 MHz, CDCl_3_): δ = 181.07, 152.61, 144.15, 140.54, 124.80, 102.92, 75.05, 72.75, 70.74, 69.74, 69.59, 69.50, 68.80, 68.54, 68.45, 63.92, 60.86, 60.46, 49.83, 31.93, 31.77, 30.38, 29.67, 29.36, 26.26, 26.13, 23.01, 22.59, 13.84, 13.76 ppm; ESI: calculated for C_80_H_138_N_10_O_31_Na [M+Na]^+^ 1757.9422, found 1757.9410 [M+Na]^+^.

### DLS and TEM Measurements

Malvern Zetasizer Nano ZS analyzer was used to determine the size of the nanostructures formed by the self‐assembly of amphiphilic architectures. Amphiphiles at a concentration of 1.0 mg mL^−1^ in water and PBS (pH 7.4) were used for DLS measurement. Samples were prepared by stirring overnight at room temperature followed by filtration through 1 µm syringe PTFE filter and then transferred to disposable UV‐transparent cuvettes for measurements. Measurements were done at least three times with 10 runs per single measurement with a duration of 60 s and the calculated mean values were taken. In order to gain further insight into the morphology and to determine the exact size of micelles, TEM measurements were done by using Thermo Scientific (FEI) Talos F200X G2 TEM operated at 200 kV accelerating voltage. The sample was prepared by applying 5 µL droplet of the aqueous solution of amphiphile onto 1 µm hole perforated carbon film covering 200 mesh grids, which was hydrophilized before applying the sample. The extra fluid on the carbon film was removed using the tip of the filter paper to create ultra‐thin layer of the sample solution covering the holes of the carbon film.

### Determination of Critical Micelle Concentration (CMC)

The critical micelle concentration of the synthesized amphiphilic architectures was determined by fluorescence encapsulation method of Nile red as a model fluorescent dye. A stock solution of 1.0 mg mL^−1^ of Nile red was prepared by dissolving in THF. The 50 µL of stock solution was added into empty vials and evaporated completely to form a thin film of Nile red. The solution of amphiphile with 5.0 mg mL^−1^ stock concentration was prepared in MilliQ water and two‐fold serial dilution was done to achieve different concentrations. The amphiphile solutions were then transferred to vials having Nile red in the same sequence and allowed to mix while stirring overnight. All the solutions were filtered through 1 µm PTFE syringe filter to remove non‐encapsulated Nile red. Fluorescence measurements of the filtered solutions were done using Agilent fluorescent spectrophotometer and plot of fluorescence maxima against varying concentration of all three amphiphile afforded the CMC values.

### Drug/Dye Encapsulation and Quantification

Navitoclax and Nile red encapsulation was done by thin film method in water. The loading experiment was carried out at a concentration of 5.0 mg mL^−1^ for all three amphiphiles using 0.5 mg of navitoclax and 0.12 mg of Nile red, respectively. The required amount of navitoclax/Nile red in methanol was taken in vial and the methanol was allowed to evaporate in order to get a uniform thin film of drug/dye at the bottom of the vial. The amphiphilic solutions of 5.0 mg mL^−1^ in water were then added to the vials having navitoclax/Nile red and stirred for overnight at room temperature. The blank samples (without amphiphilic nanocarrier) having same amount of drug/dye were also prepared for comparative studies. After overnight stirring, the non‐encapsulated drug/dye was removed by filtering twice through 1.0 µm syringe PTFE filter. For the quantification of the encapsulated navitoclax/Nile red, the loaded samples were freeze dried and re‐dissolved in HPLC grade methanol. For the Nile red encapsulated sample, UV–vis spectra were recorded on Agilent spectrophotometer using UV‐transparent disposable cuvette of 1.0 cm path length, and Beer‐Lambert's law was applied to calculate the encapsulated Nile red by using the molar extinction coefficient (ε) of 45000 M^−1^ cm^−1^ at 552 nm. The quantification of encapsulated navitoclax was carried out using HPLC (Agilent) system integrated with an internal UV detector. The mobile phase consisted of H_2_O:CH_3_CN and the flow rate was set at 1.0 mL min^−1^. The retention time of navitoclax was 5 min and the total run time was 15 min.

### Cargo Release Studies

For cargo release studies, micelles (1 mg mL^−1^) containing Nile red were dissolved in PBS and the fluorescence of Nile Red was monitored over time using the excitation wavelength of 552 nm and an emission window of 565–800 nm. This was done in both the presence and absence of the enzyme at 37° C. The β‐galactosidase enzyme from *Aspergillus oryzae* was used and purchased from Sigma Aldrich.

### Cell Culture Conditions

The A549 (human lung adenocarcinoma) cell line was obtained from the European Collection of Authenticated Cell Cultures (ECACC). The SK‐MEL‐103 (human melanoma) cancer cell line was acquired from the American Type Culture Collection (ATCC). These cell lines were maintained in Dulbecco's Modified Eagle's Medum (DMEM, Gibco, 11965092) and supplemented with 10% Fetal Bovine Serum (FBS, Gibco, 26140087).

### Senescence Induction

For senescence induction, SK‐MEL‐103 cells were supplemented with the same media containing Palbociclib (PD0332991, MCE) at 5 µm for 7 days. A549 cells were supplemented with the same media containing 15 µm Cisplatin (Stratech) for 10 days. Cisplatin (Stratech) was reconstituted in sterile PBS and Palbociclib in dimethyl sulfoxide (DMSO).

### Immuno Blotting

Cell lysis was performed using RIPA buffer (Sigma) supplemented with phosphatase inhibitors (PhosSTOP EASYpak Phosphatase Inhibitors Cocktail, Roche) and protease inhibitors (cOmplete Protease Inhibitor Cocktail, Roche). Proteins were quantified using bicinchoninic acid (BCA) assay and separated by SDS‐PAGE and transferred to polyvinylidene difluoride (PVDF) membranes (Millipore) according to standard protocols. Membranes were immunoblotted with antibodies against p21 (556430) from BD Pharmingen, phospho‐Rb (pRBS807/822) from Cell Signaling and normalized to GAPDH (ab9485) from Abcam.

### Cellular Uptake Study

Cells were seeded in a 96 well plates (Eppendorf) 3500 control cells/well and 5000 senescent cells/well in 96 well µ‐clear plates (Greiner Bio‐One). After 24 h cells were incubated with either Nile Red or branched micelles at concentrations of 10–100 µg mL^−1^ of equivalent Nile Red concentrations. Confocal images were acquired on a Leica SP5 confocal microscope using a 20X HCX PL APO 0.5 NA dry objective or a 40X HCX PL APO 1.3 NA oil immersion objective. Hoechst (Thermo Fischer)was used to specifically dye the nucleus at 5 µg mL^−1^. Lysotracker was detected by using excitation wavelength of 488 nm and with a detection window between 510 and 530 nm. Nile Red was detected with the 561 nm laser and an emission window of 595–640 nm. Images were analyzed with LAS AF Lite (Leica).

### Cytotoxicity Study

Cell viability was determined using CellTiter‐Blue assay (Promega). CellTiter Blue uses the reduction of resazurin to resorufin to measure cell viability. Cells were seeded in a 96‐well plates (Eppendorf) 3500 control cells/well and 5000 senescent cells/well. After 24 h, Micelles were added to the cells for 72 h at a range of 1 to 100 µg mL^−1^. After 72 h, 4 µL CellTiter‐Blue reagent was added to each well. After incubation for 2 h the absorbance and fluorescence were recorded for each well on the Infinite 200 Pro (Tecan) using 560/590 excitation emission. The viability studies were conducted in 4 technical replicates. The viability was calculated according to the following equation: 100 x ((Sample – Negative Control)/(Untreated Cells – Negative Control)). This was shown as percent viability.

### β‐Galactosidase In Vitro

SA‐β‐Gal staining was performed using the Senescence β‐Galactosidase Staining kit (Cell Signaling), following the manufacturer instructions. Briefly, cells were fixed at RT for 15 min with 2% formaldehyde, washed with PBS, and incubated overnight at 37 °C with the staining solution containing X‐gal in *N*,*N*‐dimethylformamide (pH 6.0). The next day cells were washed 3x with PBS for 2 min, and finally, PBS was added to the cells for imaging. Pictures were taken using a Wide Field Zeiss Axio Observer 7.

### Statistical Analysis

All analysis was performed unblinded. Statistical analyses were performed as described in the figure legend for each experiment. Statistical significance was determined by Student's t‐tests (two‐tailed) using Prism 9 software (GraphPad) as indicated. A p‐value below 0.05 was considered significant and indicated with asterisk: ^*^ *p* < 0.05, ^**^ *p* < 0.01, ^***^ *p* < 0.001, and ^****^ *p* < 0.00001.

### Figure Creation

All figures were made using Illustrator (adobe), Prism 9 software (GraphPad), as well as some picture elements generated in BioRender.

## Conflict of Interest

Andrew G Baker, Daniel MUNOZ Espin and Ljiljana Fruk are co‐founders of Senesys Bio company working on development of new generation of formulations for senolytics.

## Supporting information



Supporting Information

## Data Availability

The data that support the findings of this study are available in the supplementary material of this article.
