# Supplementary material for: Improved Therapeutic Efficiency of Senescent Cell‐specific, Galactose‐Functionalized Micelle Nanocarriers
Source: Small. 2024 Dec 18;21(7):2405732. doi: 10.1002/smll.202405732 (PMC11840467; doi:10.1002/smll.202405732)
Supplement: Supplementary file 1 — Supporting Information [file SMLL-21-2405732-s001.docx]

Supporting Information

**Galactose-functionalized amphiphilic nanocarriers for cellular senescence**

**Table of contents**

**Supplemental Figures S1 - S3.** Schematic and ^1^H and ^13^C NMR of the synthesized compound 7

**Supplemental Figures S4 - S10.** Schematic and ^1^H and ^13^C NMR of the synthesis of the ester backbone

**Supplemental Figures S11 – S16.** Schematic and ^1^H and ^13^C NMR of the synthesis of hydroxyl backbone

**Supplemental Figures S17 – S22** Schematic and ^1^H and ^13^C NMR of the synthesis alkyne backbone **Supplemental Figures S23-S28** Schematic and ^1^H and ^13^C NMR of the synthesis of protected amphiphiles

**Supplemental Figures S29-S34** Schematic and ^1^H and ^13^C NMR of the synthesis of amphiphiles

**Supplemental Table S1** Size and morphology data for linear, twinned and branched amphphiles

**Supplemental Figure S35** Higher Magnification TEM micrographs of micelles prepared using galactose-modified amphiphiles.

**Supplemental Figure S36** Loading of Nile Red and Navitoclax data for linear, twinned and branched amphphiles

**Supplemental Figure S37** TEM micrographs of micelles using galactose-modified amphiphiles loaded with Nile Red.

**Supplemental Table S2** Size and morphology data for linear, twinned and branched amphiphiles

**Supplemental Figure S38** Senescence Characterization for cells used in study

**Supplemental Figure S39** Toxicity of unloaded amphiphile micelles

**Supplemental Figure S40** Demonstration of Lysosomal delivery of the micelles in both A549 and SK-MEL-103 cell lines

**Supplemental Figure S41** Comparison of nile red single molecule and nile red delivered by micelle.

**Supplemental Figure S1**. **Synthesis of intermediate compounds to prepare amphiphiles**: i) (Boc)_2_O, ethanol, rt, 3 h; ii) NaOH, H_2_O, tetrabutylammonium iodide, propargyl bromide, rt, 50 ℃, 48 h; iii) TFA:DCM, rt, 5 h; iv) NaN_3_, DMF, 50 ℃, 16 h; v) BF_3_.Et_2_O, dry DCM, rt, 16 h.


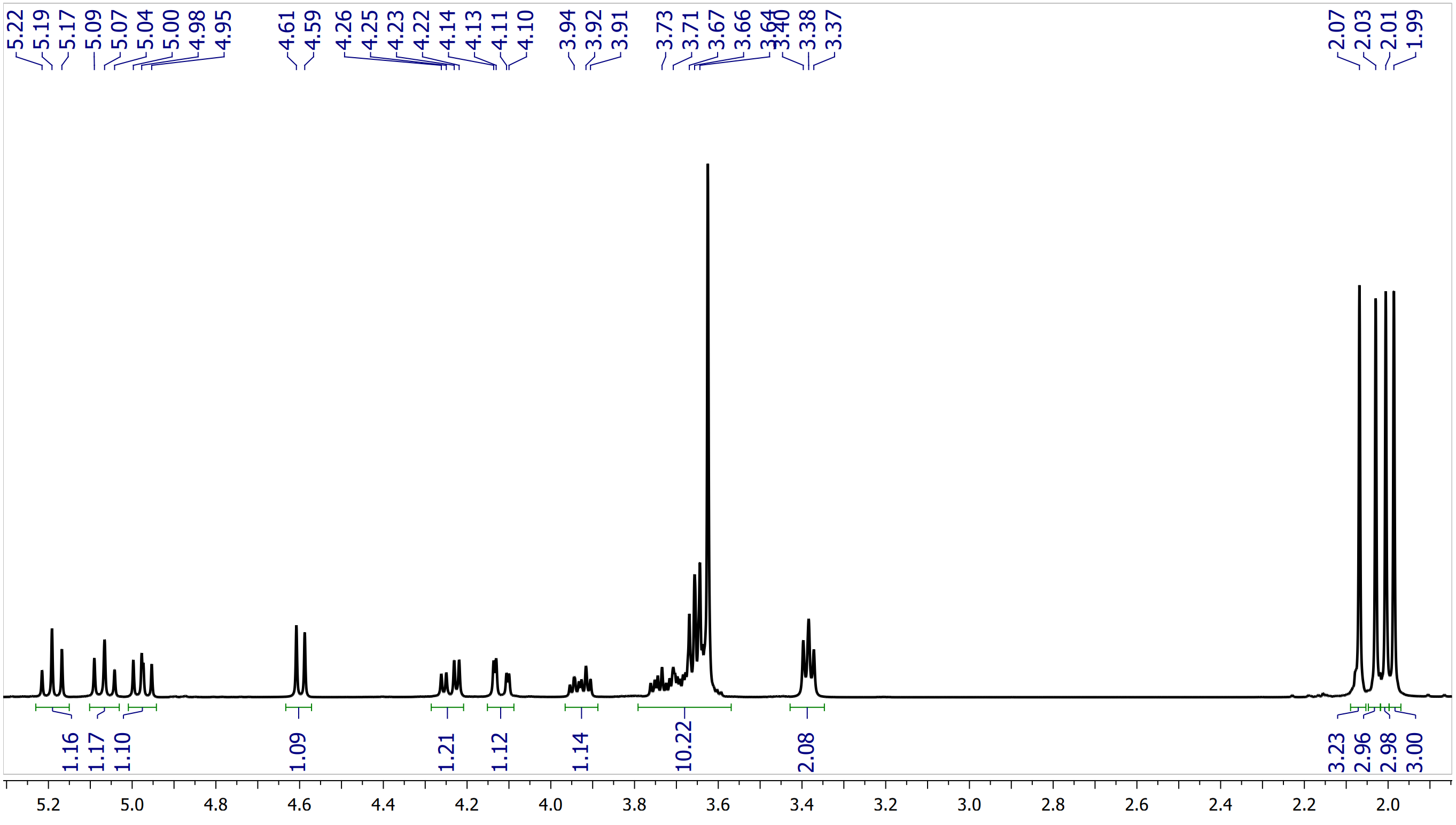


**Supplemental Figure S2**. ^1^H NMR of compound **7** in CDCl_3_.


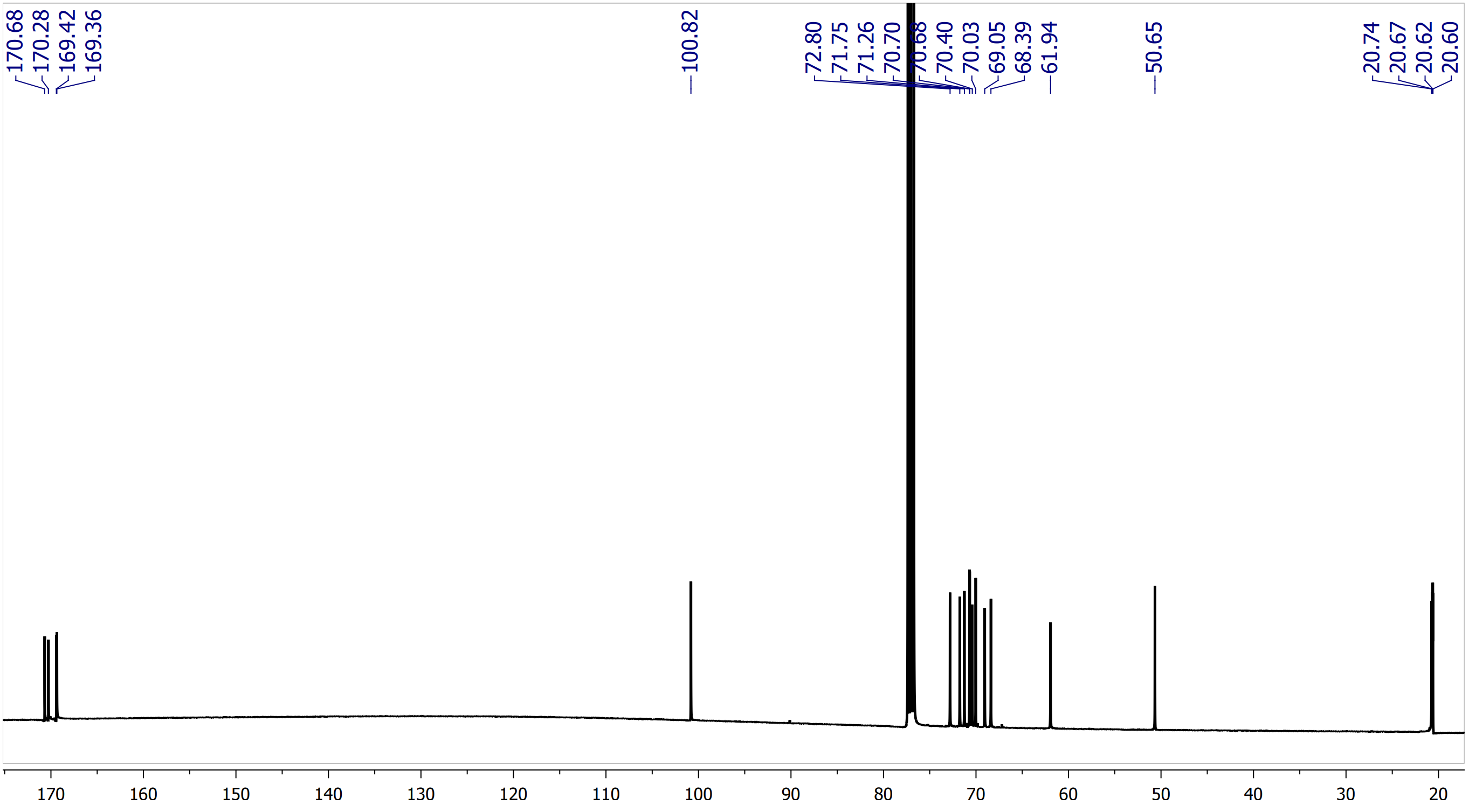


**Supplemental Figure S3**. ^13^C NMR of compound **7** in CDCl_3_.

**Detailed synthesis protocols for compounds 7-24**

Synthesis of Compound **7**

To a stirred solution of galactose pentaacetate (5 g, 12.8 mmol) and triethyleneglycol monoazide **6** (3.36 g, 19.21 mmol, 1.5 eq.) in dry DCM (50 mL) at 0 ℃, boron trifluoride etherate (7.9 mL, 64 mmol, 5 eq.) was added dropwise at under argon. The reaction mixture was stirred at rt for 24 h. Upon completion of the reaction, the mixture was poured in saturated aq. NaHCO_3_ sol (400 mL) and extracted with DCM (2x200 mL). The combined organic layer was dried over anhydrous Na_2_SO_4_ and concentrated under reduced pressure. The crude product obtained was purified by column chromatography (Hexane/EtOAc) affording the desired compound **7** (yield 65%). ^1^H NMR (400 MHz, CDCl_3_): δ = 5.19 (t, *J* = 9.5 Hz, 1H), 5.07 (t, *J* = 9.7 Hz, 1H), 5.01-4.95 (m, 1H), 4.60 (d, *J* = 8.0 Hz, 1H), 4.24 (dd, *J* = 12.3, 4.7 Hz, 1H), 4.12 (dd, *J* = 12.3, 2.3 Hz, 1H), 3.94-391 (m, 1H), 3.78-3.58 (m, 8H), 3.43-3.34 (m, 2H), 2.07 (s, 3H), 2.03 (s, 3H), 2.01 (s, 3H), 1.99 (s, 3H); ^13^C NMR (100.5 MHz, CDCl_3_): δ = 170.68, 170.28, 169.42, 169.36, 100.82, 72.80, 71.75, 71.26, 70.70, 70.68, 70.40, 70.03, 69.05, 68.39, 61.94, 50.65, 20.74, 20.67, 20.62, 20.60.

Synthesis of Compound **9/15/21**

To a stirred mixture of **8**/**14**/**20** (1.0 g, 1 eq.) and K_2_CO_3_ (3/6/9 eq.) in acetonitrile (50/75/100 mL), 1-bromooctane was added dropwise over a period of 30 min. The resulting mixture was stirred at 50 ℃ for 16 h. On completion of the reaction, acetonitrile was removed under reduced pressure and the residue was extracted with DCM (2 x 100 mL). The combined organic layer was washed with saturated NaHCO_3_ (2 x 100 mL) and brine (2 x 100 mL) and dried over Na_2_SO_4_. The crude product obtained after concentrated under reduced pressure was purified by column chromatography (Hexane/EtOAc) affording the desired alkylated derivatives (**9**/**15**/**21**).

Compound **9**

Obtained in 80% yield; ^1^H NMR (400 MHz, CDCl_3_): δ = 7.97 (d, J = 9.0 Hz, 2H), 6.89 (d, J = 9.0 Hz, 2H), 3.98 (t, J = 6.6 Hz, 2H), 3.86 (s, 3H), 1.81-1.74 (m, 2H), 1.48-1.41 (m, 2H), 1.36-1.27 (m, 8H), 0.88 (t, J = 6.9 Hz, 3H); ^13^C NMR (100.5 MHz, CDCl_3_): δ = 166.86, 162.94, 131.53, 122.27, 114.02, 68.17, 51.77, 31.80, 29.33, 29.22, 29.11, 25.99, 22.65, 14.09 ppm.

Compound **15**

Obtained in 75% yield; ^1^H NMR (400 MHz, CDCl_3_): δ = 7.14 (d, J = 2.3 Hz, 2H), 6.61 (t, J = 2.2 Hz, 1H), 3.94 (t, J = 6.6 Hz, 4H), 3.86 (s, 3H), 1.79-1.71 (m, 4H), 1.46-1.39 (m, 4H), 1.32-1.27 (m, 16H), 0.87 (t, J = 6.8 Hz, 6H); ^13^C NMR (100.5 MHz, CDCl_3_): δ = 166.86, 160.12, 131.75. 107.54, 106.44, 68.21, 52.04, 31.82, 29.34, 29.25, 29.18, 26.02, 22.66, 14.06 ppm.

Compound **21**

Obtained in 73% yield; ^1^H NMR (400 MHz, CDCl_3_): δ = 7.24 (s, 2H), 4.02-3.98 (m, 6H), 3.86 (s, 3H), 1.83-1.71 (m, 6H), 1.50-1.43 (m, 6H), 1.36-1.27 (m, 24H), 0.87 (t, J = 6.9 Hz, 9H); ^13^C NMR (100.5 MHz, CDCl_3_): δ = 166.83, 152.78, 142.31, 124.63, 107.90, 73.41, 69.08, 51.99, 31.89, 31.82, 30.33, 29.50, 29.36, 29.34, 29.29, 29.28, 26.07, 26.05, 22.66, 14.06 ppm.

Synthesis of Compound **10/16/22**

In a 100 mL round bottom, an aqueous solution of sodium hydroxide (1N aq. NaOH, 2 mL) was added to a solution of compound **9**/**15**/**21** (1.0 g) in THF (25 mL). The resulting mixture was stirred at rt for 5 h. On completion of the reaction, the solution was neutralized with 1N HCl. Solvent was removed and the obtained residue was washed with hexane and dried to obtain the desired compound **10**/**16**/**22**.

Compound **10**

Obtained in 84% yield; ^1^H NMR (400 MHz, DMSO-d_6_): δ = 7.86 (d, J = 8.3 Hz, 2H), 6.98 (d, J = 8.2 Hz, 2H), 4.01 (t, J = 6.2 Hz, 2H), 1.74-1.68 (m, 2H), 1.42-1.26 (m, 8H), 0.86 (t, J = 6.5 Hz, 3H); ^13^C NMR (100.5 MHz, DMSO-d_6_): δ = 167.59, 162.54, 131.72, 123.90, 114.55, 68.17, 31.69, 29.17, 29.11, 29.00, 25.91, 22.54, 14.42 ppm.

Compound **16**

Obtained in 86% yield; ^1^H NMR (400 MHz, CDCl_3_): δ =6.95 (bs, 2H), 6.37 (bs, 1H), 3.68 (t, 4H), 159-151 (m, 4H), 1.31-1.24 (m, 20H), 0.88 (t, J = 6.9 Hz, 6H); ^13^C NMR (100.5 MHz, CDCl_3_): δ = 173.26, 159.71, 134.77, 107.24, 105.96, 67.89, 31.94, 29.61, 29.34, 26.13, 22.70, 14.10 ppm.

Compound **22**

Obtained in 86% yield; ^1^H NMR (400 MHz, CDCl_3_): δ = 7.33 (s, 2H), 4.06-4.01 (m, 6H), 1.86-1.72 (m, 6H), 1.52-1.44 (m, 6H), 1.38-1.29 (m, 24H), 0.89 (m, 9H); ^13^C NMR (100.5 MHz, CDCl_3_): δ = 172.01, 152.83, 143.12, 123.63, 108.51, 73.55, 69.17, 31.90, 31.84, 30.33, 29.51, 29.37, 29.35, 29.29, 29.27, 26.08, 26.03, 22.70, 22.68, 14.11 ppm.

Synthesis of Compound **11/17/23**

To a stirred solution of **10**/**16**/**22** (500 mg, 1 eq.) in DMF (30 mL), EDC.HCl (2 eq.), HOBt (1 eq.) and DIPEA (2 eq.) were added. The mixture was stirred at rt for 20 min followed by the portion-wise addition of compound **4a**/**4b**/**4c** (1.2 eq.). After 16 h of stirring, the completion of the reaction was checked by TLC. On completion of the reaction, DMF was removed under reduced pressure and the mixture was extracted with DCM (2 x 100 mL). The combined organic layer was washed with saturated NaHCO_3_ (2 x 100 mL) and brine (2 x 100 mL) and dried over Na_2_SO_4_. The crude product obtained after concentrated under reduced pressure was purified by column chromatography (Hexane/EtOAc) affording the desired alkylated derivatives (**11**/**17**/**23**).

Compound **11**

Obtained in 73% yield; ^1^H NMR (400 MHz, CDCl_3_): δ = 7.78 (d, *J* = 8.8 Hz, 2H), 6.88 (d, *J* = 8.8 Hz, 2H), 6.84 (bs, 1H), 4.21 (dd, *J* = 5.1, 2.5 Hz, 2H), 3.97 (t, *J* = 6.6 Hz, 2H), 2.22 (t, *J* = 2.5 Hz, 1H), 1.79-1.72 (m, 2H), 1.46-1.39 (m, 2H), 1.36-1.23 (m, 8H), 0.86 (t, *J* = 6.8 Hz, 3H); ^13^C NMR (100.5 MHz, CDCl_3_): δ = 166.92, 161.95, 128.97, 125.69, 114.17, 79.90, 71.45, 68.16, 31.78, 29.64, 29.32, 29.21, 29.11, 25.98, 22.64, 14.09 ppm.

Compound **17**

Obtained in 69% yield; ^1^H NMR (400 MHz, CDCl_3_): δ = 6.87 (d, *J* = 2.1 Hz, 2H), 6.55 (t, *J* = 2.0 Hz, 1H), 6.47 (d, *J* = 8.4 Hz, 1H), 4.48-4.41 (m, 1H), 4.23-4.14 (m, 4H), 3.95 (t, *J* = 6.6 Hz, 4H), 3.75 (dd, *J* = 9.4, 4.3 Hz, 2H), 3.66 (dd, *J* = 9.4, 5.8 Hz, 2H), 2.43 (t, *J* = 2.3 Hz, 1H), 1.79-1.72 (m, 4H), 1.46-1.39 (m, 4H), 1.32-1.23 (m, 16H), 0.87 (t, *J* = 6.7 Hz, 6H); ^13^C NMR (100.5 MHz, CDCl_3_): δ = 167.00, 160.31, 136.38, 105.42, 104.31, 79.46, 74.78, 68.30, 68.16, 58.49. 48.58, 29.32, 29.22, 29.18, 26.01, 22.65, 14.10 ppm.

Compound **23**

Obtained in 75% yield; ^1^H NMR (400 MHz, CDCl_3_): δ = 6.94 (s, 2H), 6.39 (s, 1H), 4.17 (d, *J* = 2.4 Hz, 6H), 4.04-3.93 (m, 12H), 2.43 (t, *J* = 2.3 Hz, 3H), 1.86-1.66 (m, 6H), 1.48-1.41 (m, 6H), 1.35-1.27 (m, 24H), 0.87 (t, *J* = 6.5 Hz, 9H); ^13^C NMR (100.5 MHz, CDCl_3_): δ = 167.22, 152.95, 141.16, 129.97, 105.83, 79.62, 74.64, 73.47, 69.34, 68.63, 59.42, 58.70, 31.89, 31.82, 30.28, 29.51, 29.35, 29.34, 28.28, 26.06, 22.66, 14.10 ppm.

Synthesis of Compound **12/18/24**

To a solution of compound **11**/**17**/**23** (150 mg, 1 eq.) and compound **7** (1/2/3 eq.) in a THF/H_2_O mixture (3:1, 20 mL), sodium ascorbate (0.2/0.4/0.6 eq.) and CuSO_4_.5H_2_O (0.2/0.4/0.6 eq.) were added. The resulting solution was stirred at 50 ℃ for 16 h. On completion of the reaction, THF was removed under reduced pressure and the mixture was extracted with DCM (2 x 50 mL). The combined organic layer was washed with saturated aq. NH_4_Cl (2 x 50 mL) and brine solution (2 x 50 mL). The resolution solution was then dried over anhydrous Na_2_SO_4_ and concentrated under reduced pressure. The crude product obtained was purified by column chromatography (DCM/MeOH) affording the desired compound **12/18/24**.

Compound **12**

Obtained in 78% yield; ^1^H NMR (400 MHz, CDCl_3_): δ = 7.76 (s, 1H), 7.74 (d, *J* = 8.7 Hz, 2H), 7.20 (bs, 1H), 6.85 (d, *J* = 8.7 Hz, 2H), 5.34 (d, *J* = 3.3 Hz, 1H), 5.26 (s, 1H), 5.15 (dd, *J* = 10.4, 8.0 Hz, 1H), 5.00 (dd, *J* = 10.5, 3.4 Hz, 1H), 4.66 (d, *J* = 5.1 Hz, 2H), 4.54-4.47 (m, 3H), 4.15-4.06 (m, 2H), 3.95-3.89 (m, 3H), 3.83 (t, *J* = 5.1 Hz, 2H), 3.70-3.64 (m, 1H), 3.57-3.54 (m, 6H), 2.09 (s, 3H), 1.99 (s, 3H), 1.98 (s, 3H), 1.94 (s, 3H), 1.77-1.70 (m, 2H), 1.44-1.37 (m, 2H), 1.32-1.18 (m, 8H), 0.84 (t, *J* = 6.7 Hz, 3H); ^13^C NMR (100.5 MHz, CDCl_3_): δ = 170.37, 170.23, 170.12, 169.47, 166.89, 161.82, 128.85, 126.01, 114.13, 101.26, 70.84, 70.60, 70.57, 70.53, 70.17, 69.36, 69.13, 68.82, 68.13, 67.05, 61.24, 53.47, 50.25, 35.18, 31.75, 29.29, 29.18, 29.10, 25.95, 22.61, 20.75, 20.66, 20.63, 20.52, 14.07 ppm.

Compound **18**

Obtained in 73% yield; ^1^H NMR (400 MHz, CDCl_3_): δ = 7.72 (s, 2H), 6.86 (d, *J* = 2.0 Hz, 2H), 6.74 (d, *J* = 8.1 Hz, 2H), 6.52 (t, *J* = 1.7 Hz, 1H), 5.36 (d, *J* = 3.3 Hz, 2H), 5.16 (dd, *J* = 10.4, 8.0 Hz, 2H), 4.99 (dd, *J* = 10.5, 3.3 Hz, 2H), 4.63 (s, 4H), 4.53-4.51 (m, 6H), 4.39 (bs, 1H), 4.16-4.07 (m, 4H), 3.96-3.83 (m, 12H), 3.74-3.55 (m, 18H), 2.11 (s, 6H), 2.01 (s, 6H), 2.00 (s, 6H), 1.95 (s, 6H), 1.77-1.70 (m, 4H), 1.45-1.38 (m, 4H), 1.35-1.22 (m, 16H), 0.86 (t, *J* = 6.8 Hz, 6H); ^13^C NMR (100.5 MHz, CDCl_3_): δ = 174.29, 170.38, 170.23, 170.12, 169.43, 167.05, 160.27, 144.43, 136.26, 123.85, 105.44, 104.42, 101.30, 70.84, 70.62, 70.57, 70.50, 70.16, 69.39, 69.13, 68.78, 68.48, 68.30, 67.02, 64.35, 61.22, 50.22, 49.06, 31.79, 29.32, 29.21, 26.00, 22.63, 20.76, 20.67, 20.57, 14.09 ppm.

Compound **24**

Obtained in 70% yield; ^1^H NMR (400 MHz, CDCl_3_): δ = 7.66 (s, 3H), 6.96 (s, 2H), 6.72 (bs, 2H), 5.35 (d, *J* = 3.1 Hz, 2H), 5.27 (bs, 3H), 5.15 (dd, *J* = 10.3, 8.1 Hz, 2H), 4.99 (dd, *J* = 10.4, 3.3 Hz, 2H), 4.59 (s, 4H), 4.52-4.48 (m, 8H), 4.39 (bs, 1H), 4.15-4.07 (m, 6H), 3.99-3.82 (m, 24H), 3.69-3.65 (m, 3H), 3.59-3.54 (m, 18H), 2.11 (s, 9H), 2.00 (s, 9H), 2.00 (s, 9H), 1.94 (s, 9H), 1.79-1.67 (m, 6H), 1.46-1.40 (m, 6H), 1.33-1.21 (m, 24H), 0.85 (t, *J* = 6.8 Hz, 9H); ^13^C NMR (100.5 MHz, CDCl_3_): δ = 170.36, 170.22, 170.10, 169.42, 167.29, 152.94, 144.48, 140.94, 129.93, 123.74, 105.76, 101.30, 73.43, 70.85, 70.63, 70.56, 70.48, 70.16, 69.40, 69.31, 69.10, 68.87, 68.78, 67.03, 64.59, 61.21, 60.09, 53.45, 50.14, 31.87, 31.81, 30.30, 29.51, 29.40, 29.38, 29.34, 29.28, 26.12, 26.06, 22.64, 20.75, 20.66, 20.56, 14.08 ppm.

**Branched amphiphile**


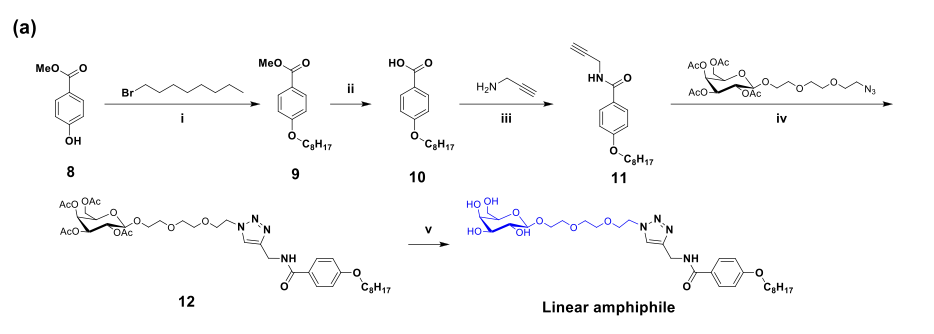


a


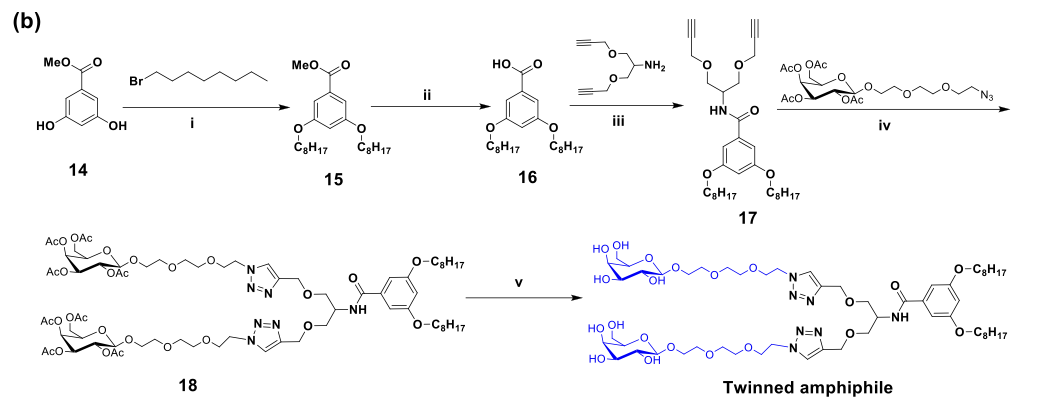


b


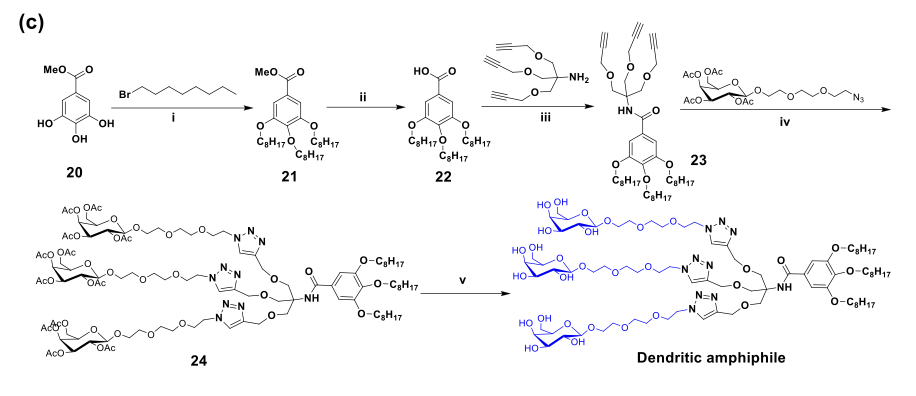


c

**Supplemental Figure S4. Overview of amphiphile synthesis.** Synthesis of Linear amphphiles: i) K_2_CO_3_, acetonitrile, 50 ℃, 16 h; ii) 2N aq. NaOH, THF, rt, 5 h; iii) EDC.HCl, HOBt, DIPEA, DMF, rt, 16 h; iv) CuSO_4_.5H_2_O, sodium ascorbate, THF, H_2_O, 50 ℃, 16 h; v) 2N aq. NaOH, THF, rt, 5 h. **b)** Synthesis of Twinned amphphiles: i) K_2_CO_3_, acetonitrile, 50 ℃, 16 h; ii) 2N aq. NaOH, THF, rt, 5 h; iii) EDC.HCl, HOBt, DIPEA, DMF, rt, 16 h; iv) CuSO_4_.5H_2_O, sodium ascorbate, THF, H2O, 50 ℃, 16 h; v) 2N aq. NaOH, THF, rt, 5 h. c) Synthesis of Branched amphphiles: i) K_2_CO_3_, acetonitrile, 50 ℃, 16 h; ii) 2N aq. NaOH, THF, rt, 5 h; iii) EDC.HCl, HOBt, DIPEA, DMF, rt, 16 h; iv) CuSO_4_.5H_2_O, sodium ascorbate, THF, H_2_O, 50 ℃, 16 h; v) 2N aq. NaOH, THF, rt, 5 h.


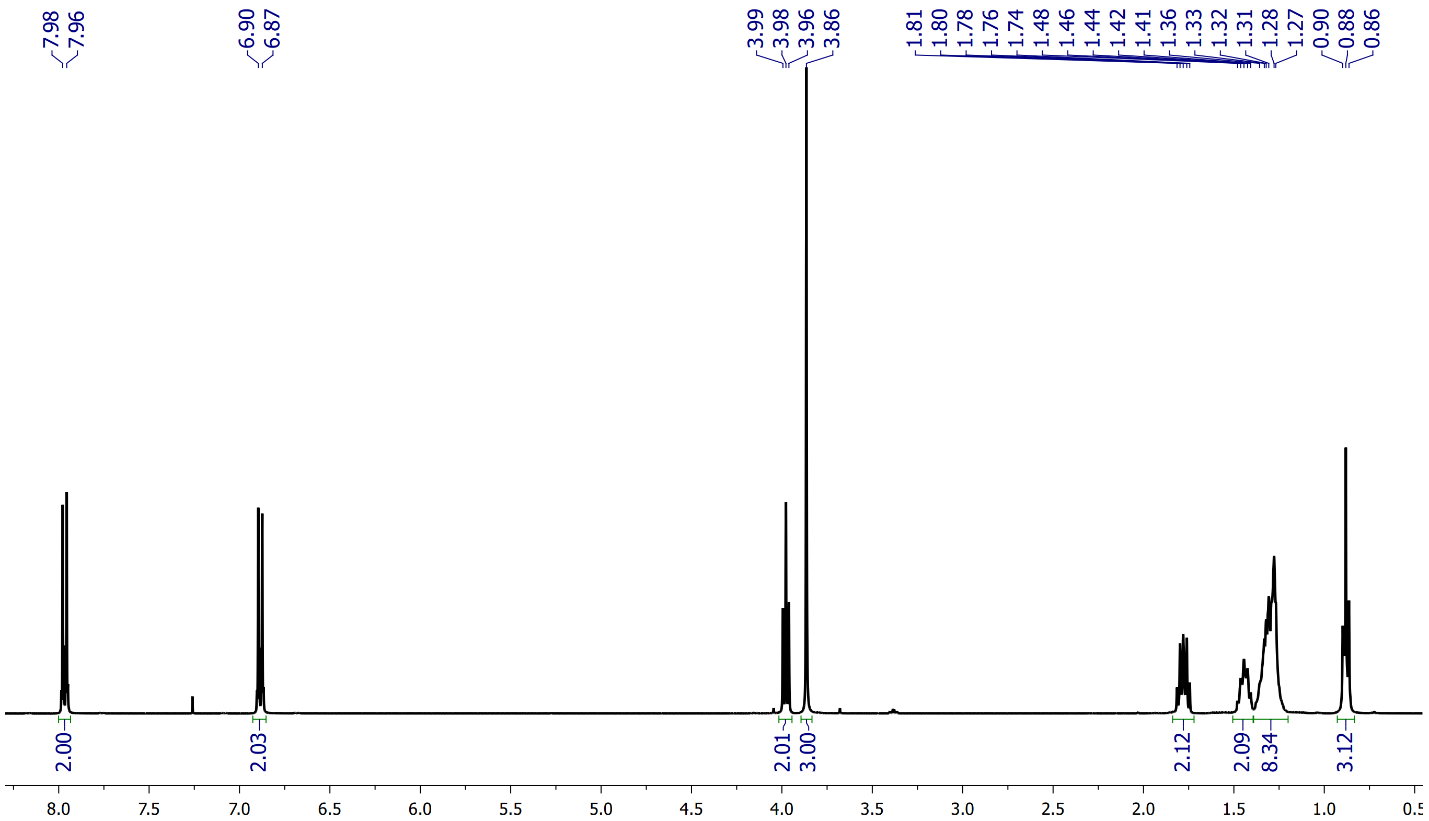


**Supplemental Figure S5**. ^1^H NMR of compound **9** in CDCl_3_.


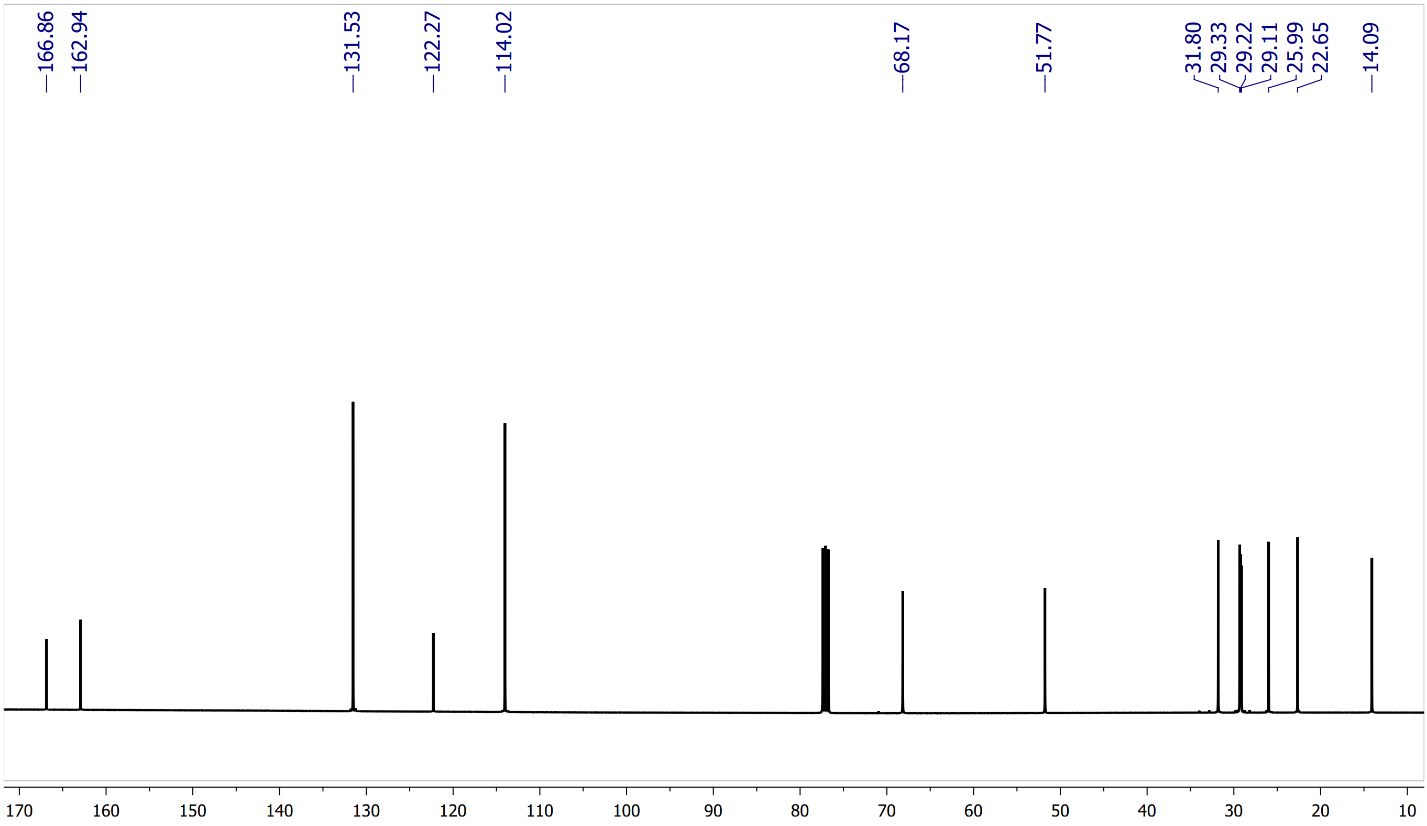


**Supplemental Figure S6**. ^13^C NMR of compound **9** in CDCl_3_.


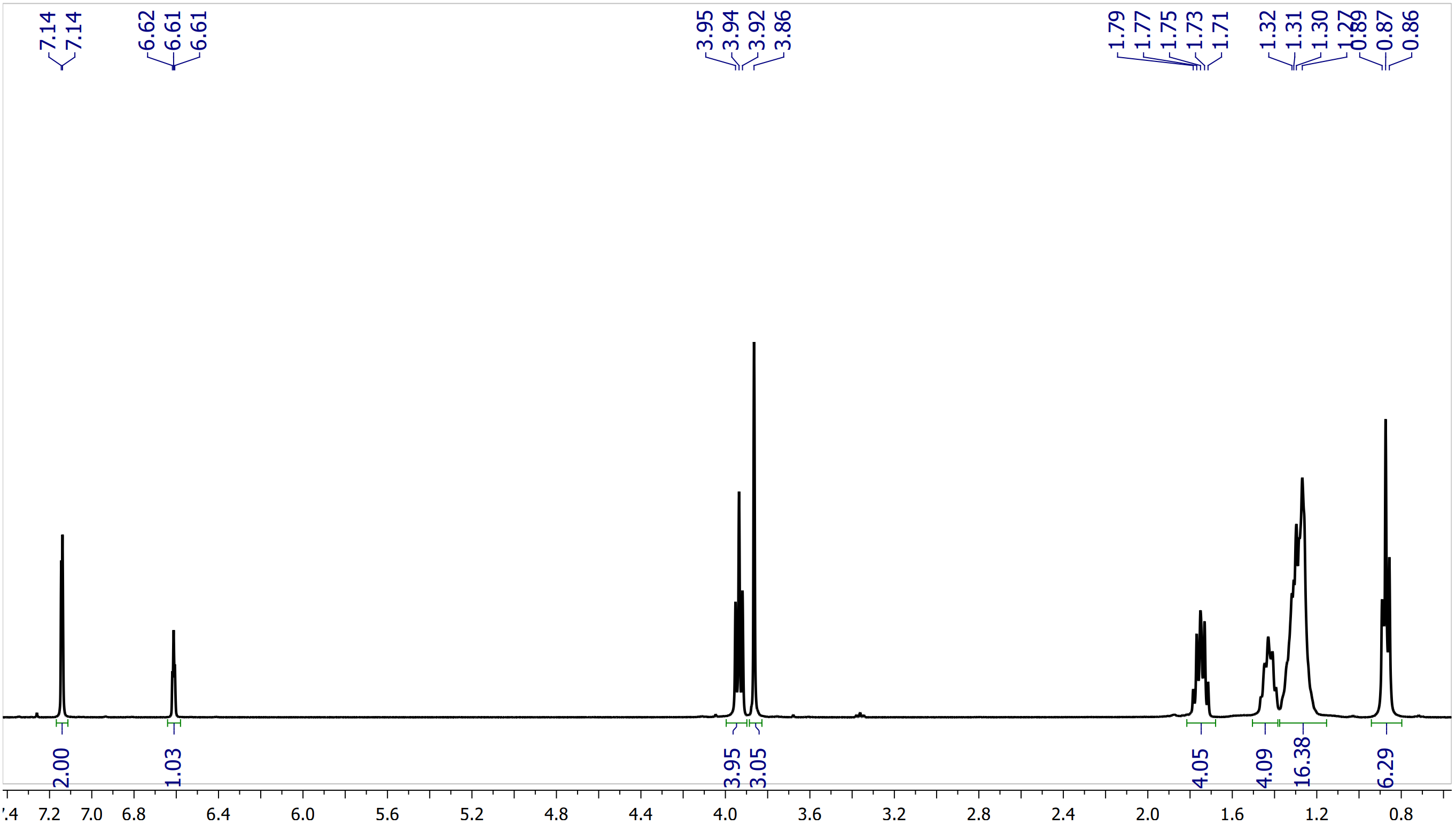


**Supplemental Figure S7**. ^1^H NMR of compound **15** in CDCl_3_.


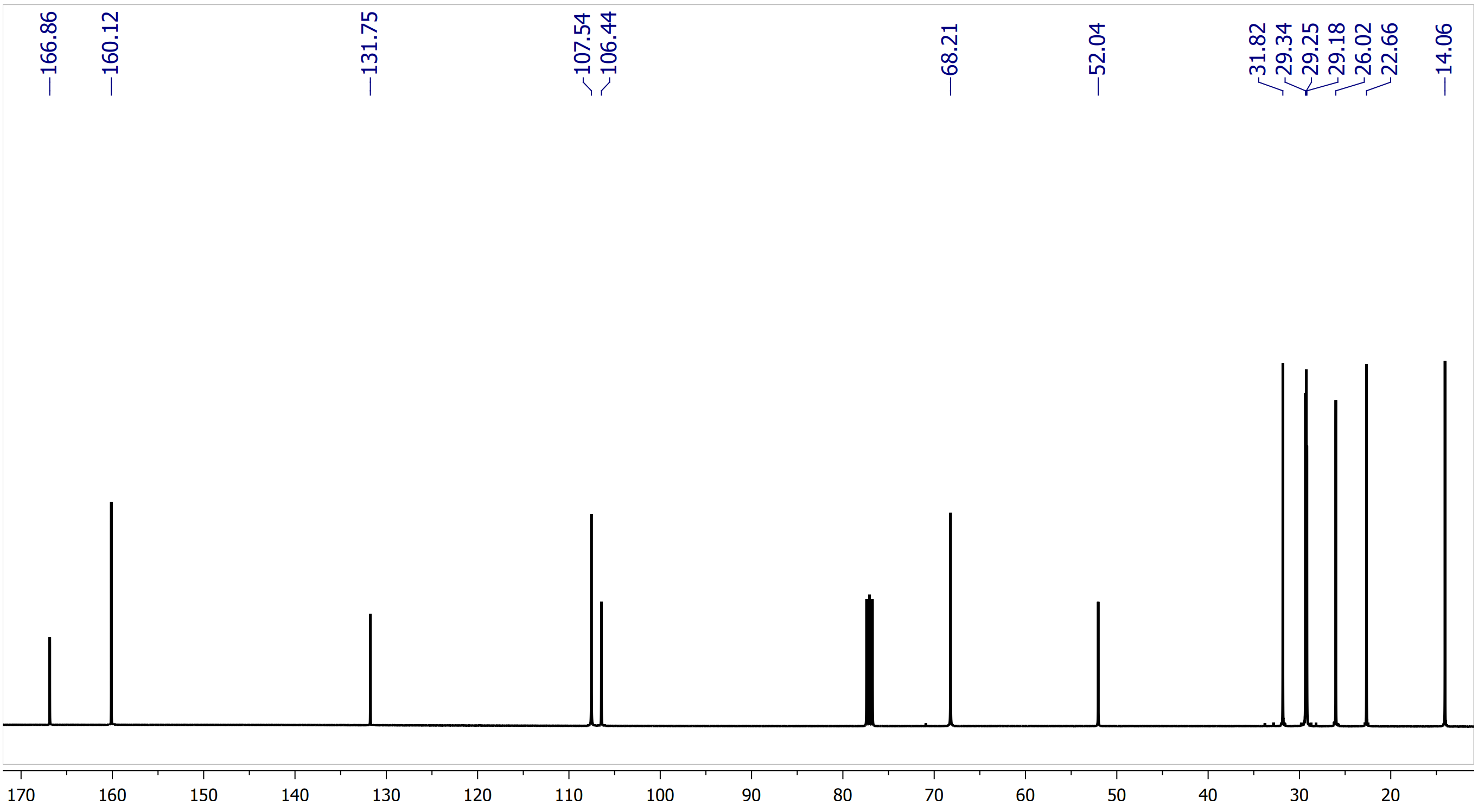


**Supplemental Figure S8**. ^13^C NMR of compound **15** in CDCl_3_.


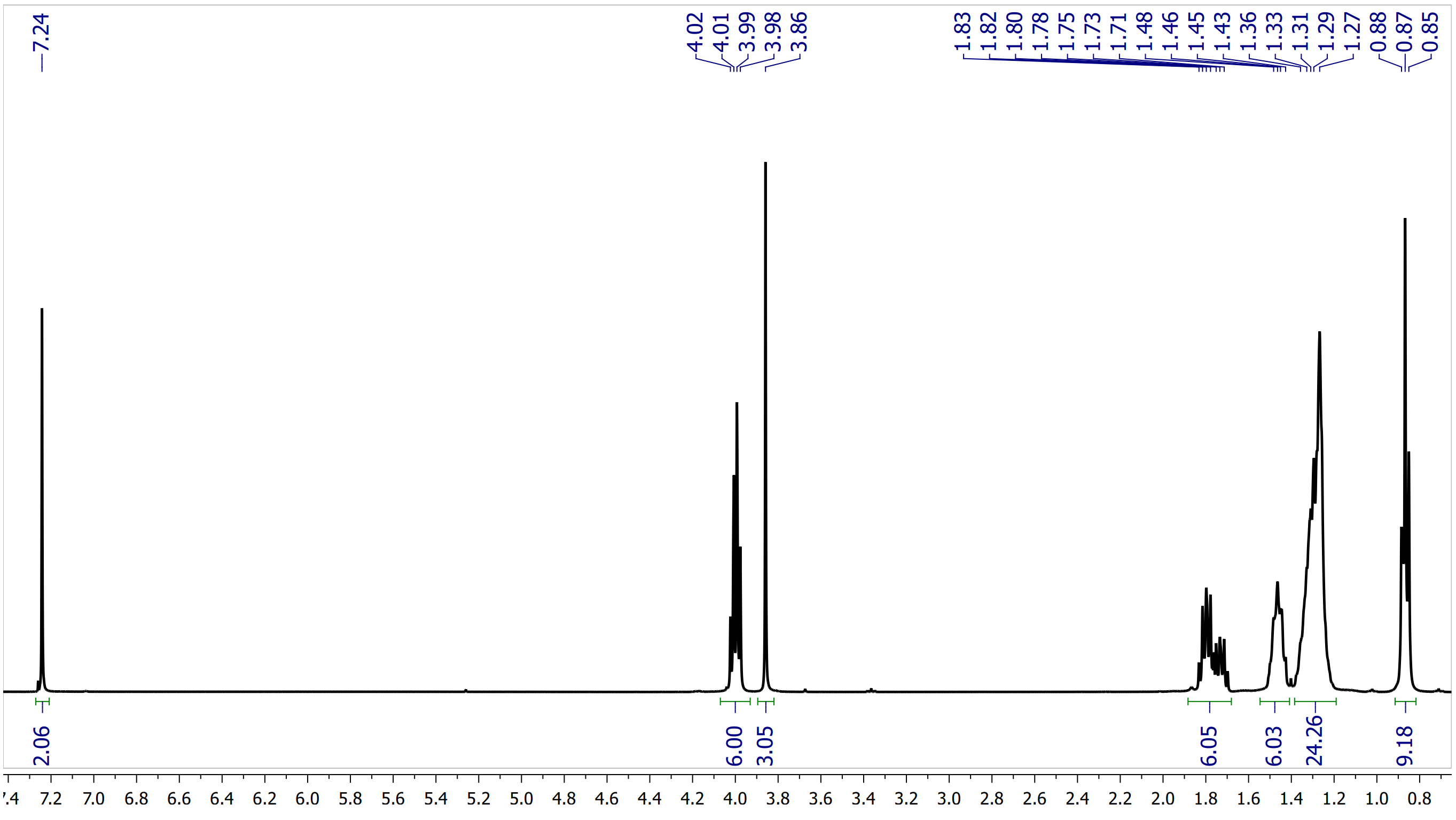


**Supplemental Figure S9**. ^1^H NMR of compound **21** in CDCl_3_.


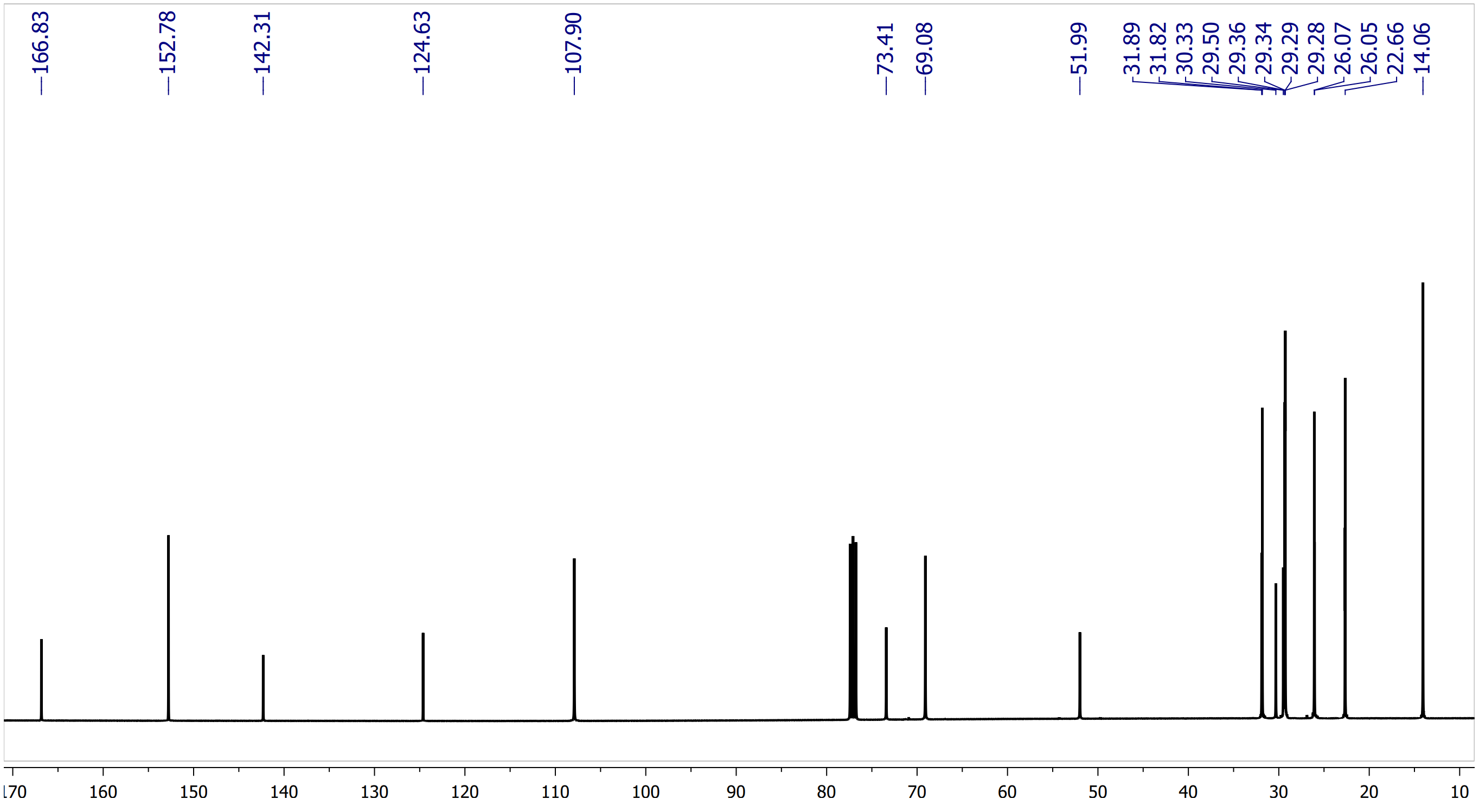


**Supplemental Figure S10**. ^13^C NMR of compound **21** in CDCl_3_.


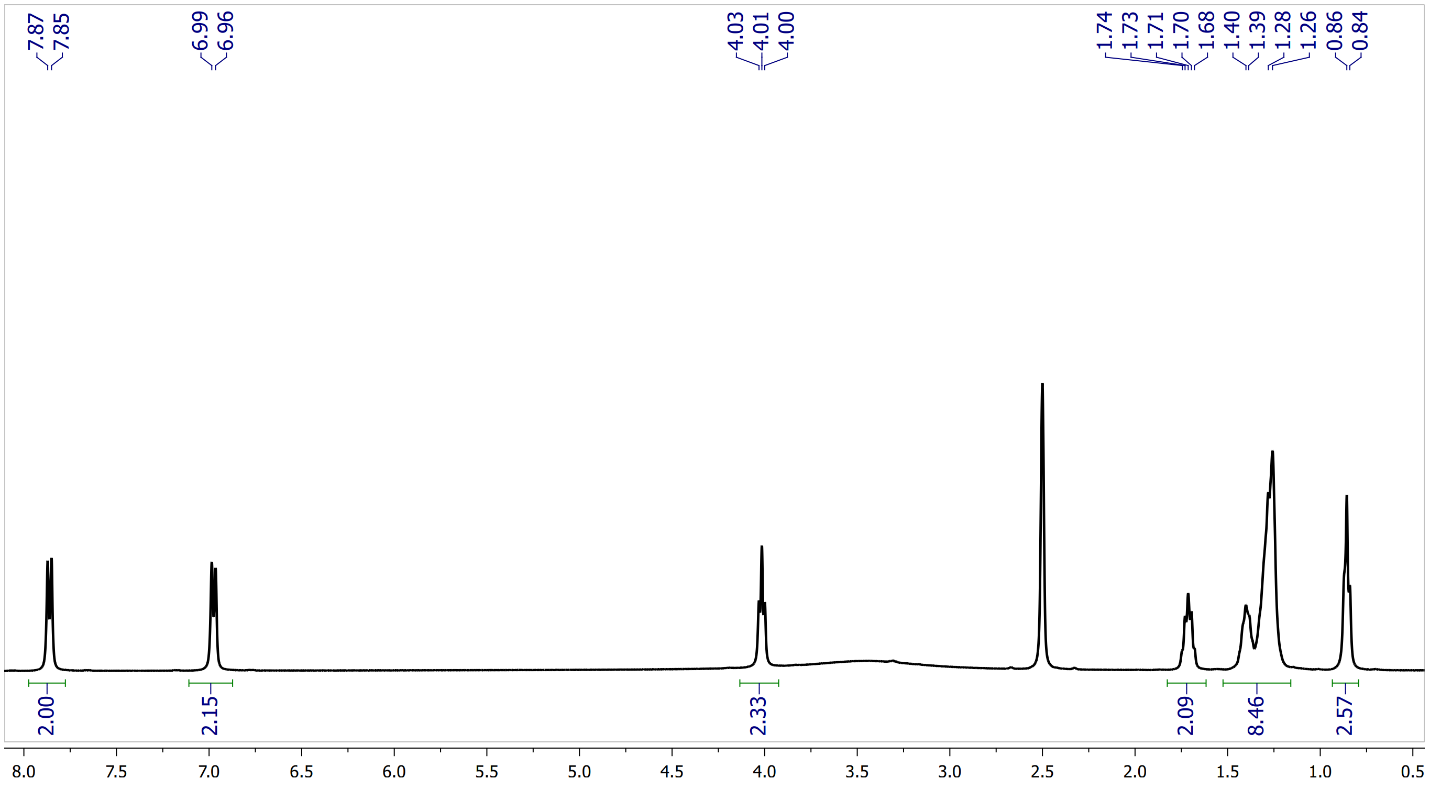


**Supplemental Figure S11**. ^1^H NMR of compound **10** in DMSO-d_6_.


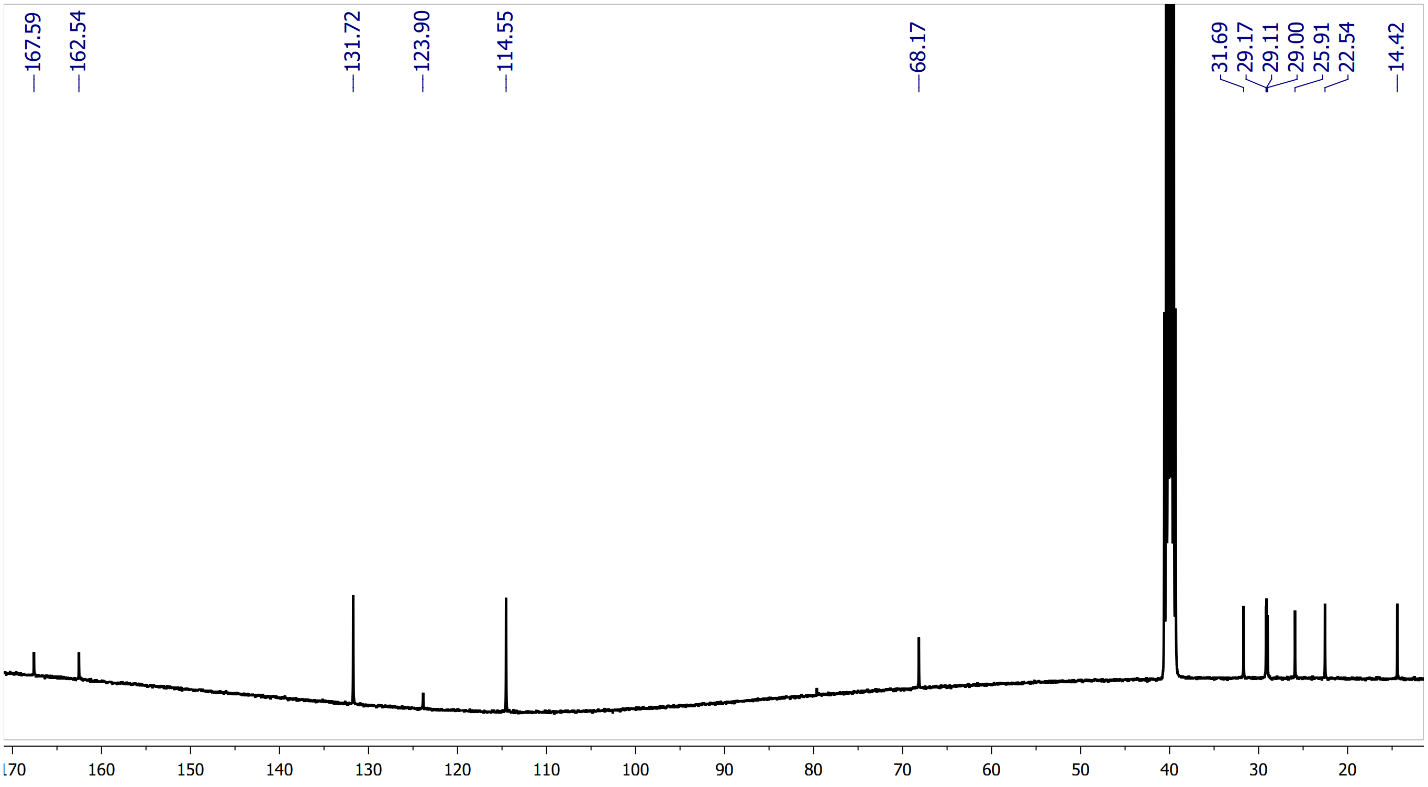


**Supplemental Figure S12**. ^13^C NMR of compound **10** in DMSO-d_6_.


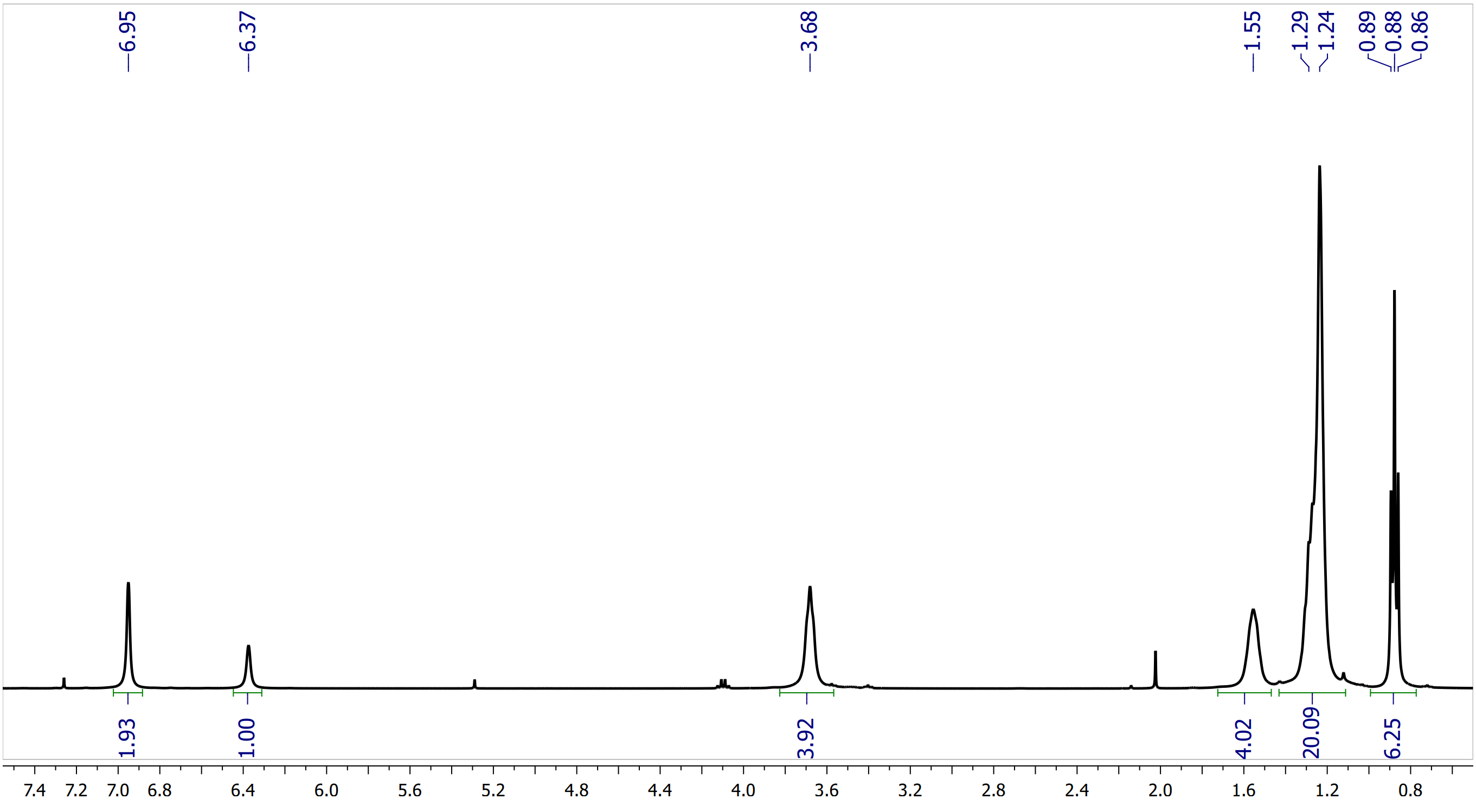


**Supplemental Figure S13**. ^1^H NMR of compound **16** in CDCl_3_.


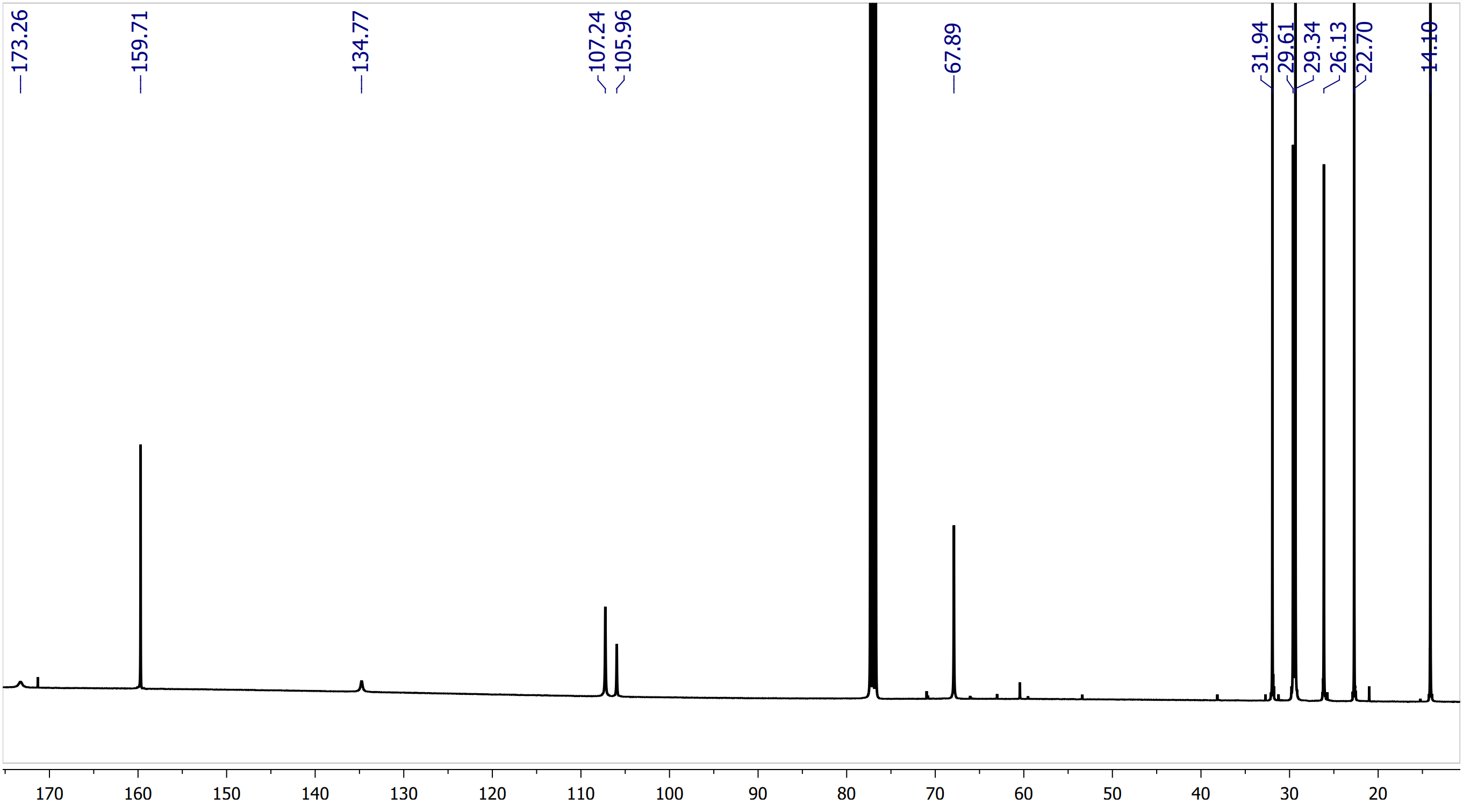


**Supplemental Figure S14**. ^13^C NMR of compound **16** in CDCl_3_.


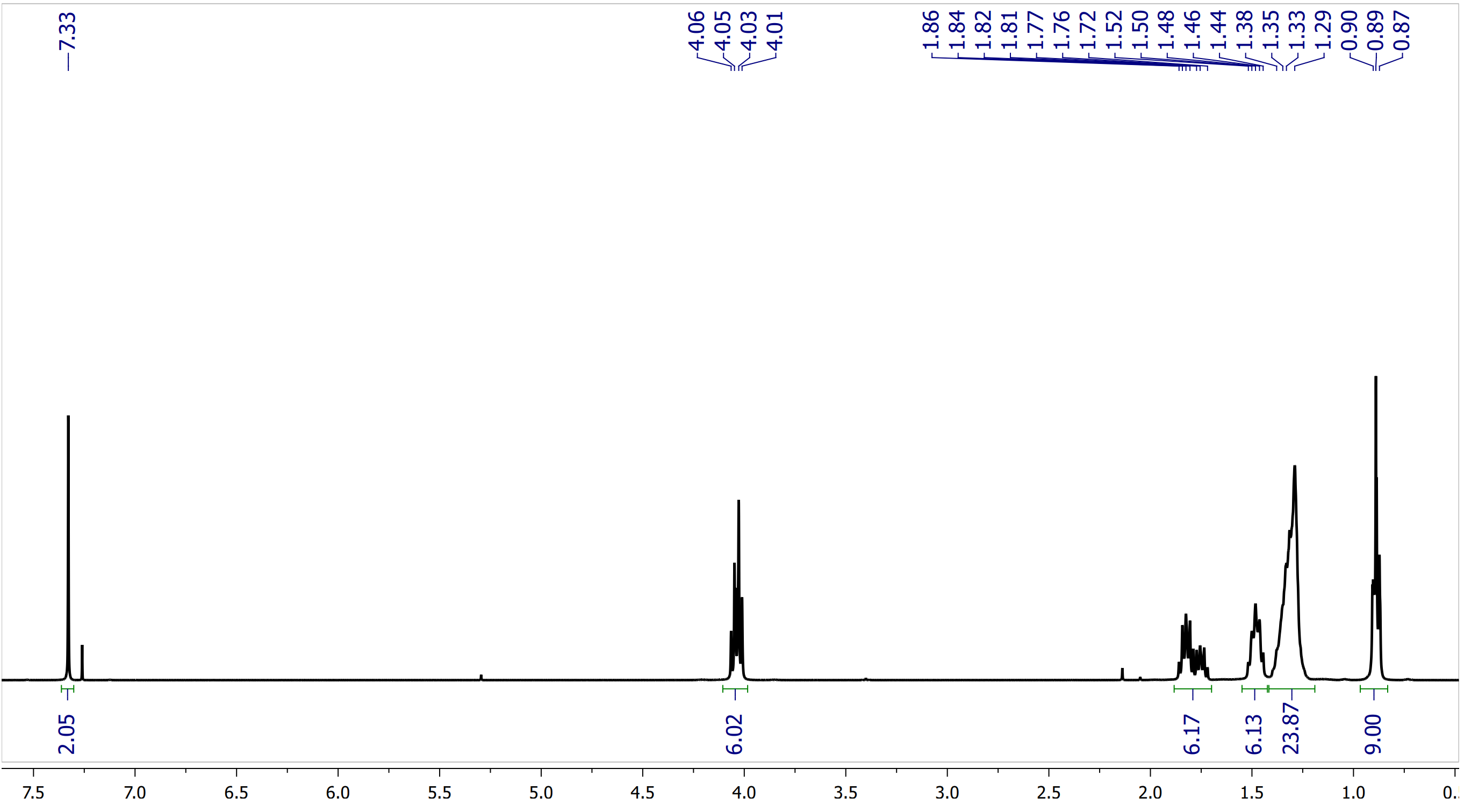


**Supplemental Figure S15**. ^1^H NMR of compound **22** in CDCl_3_.


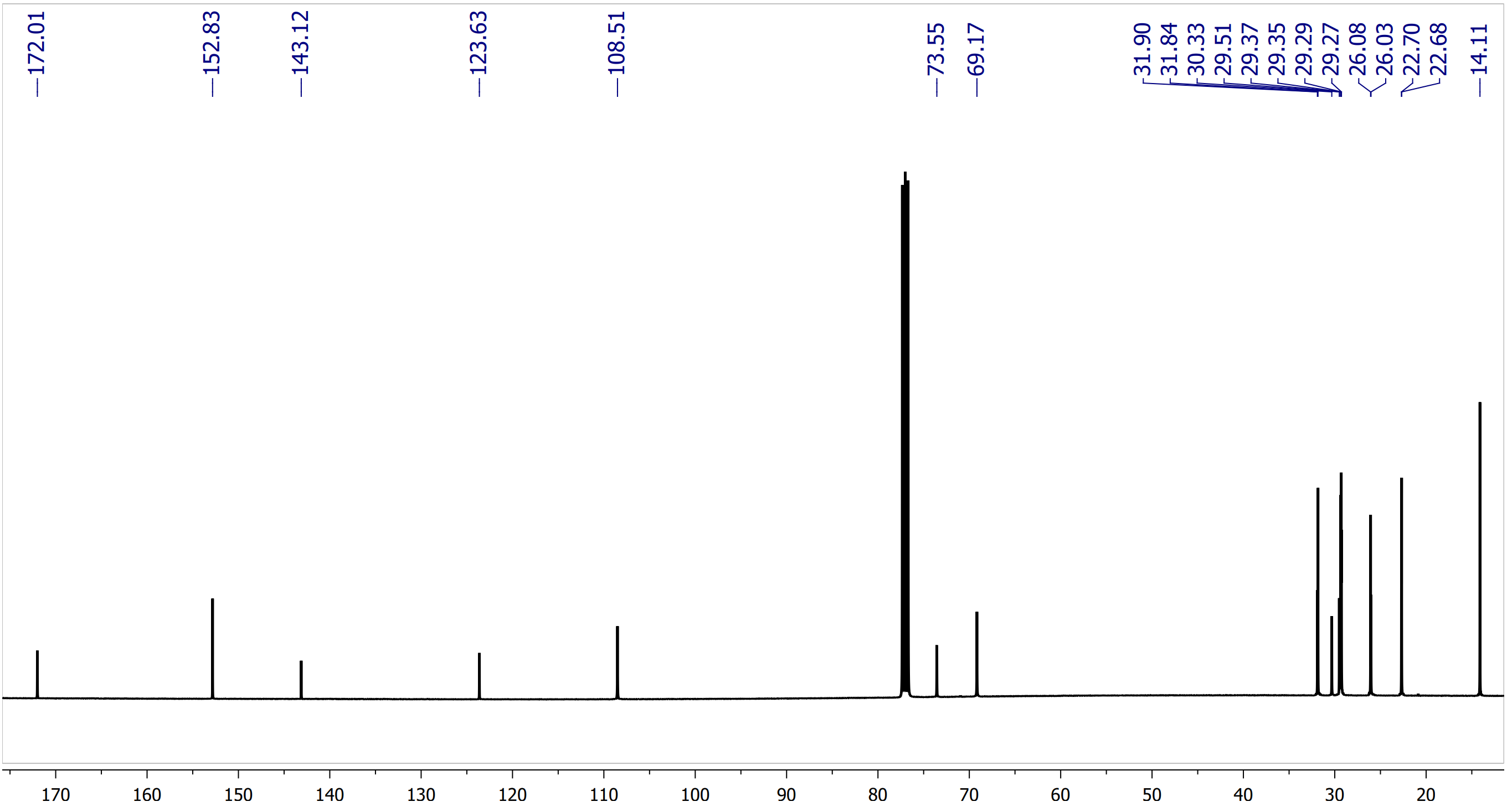


**Supplemental Figure S16**. ^13^C NMR of compound **22** in CDCl_3_.


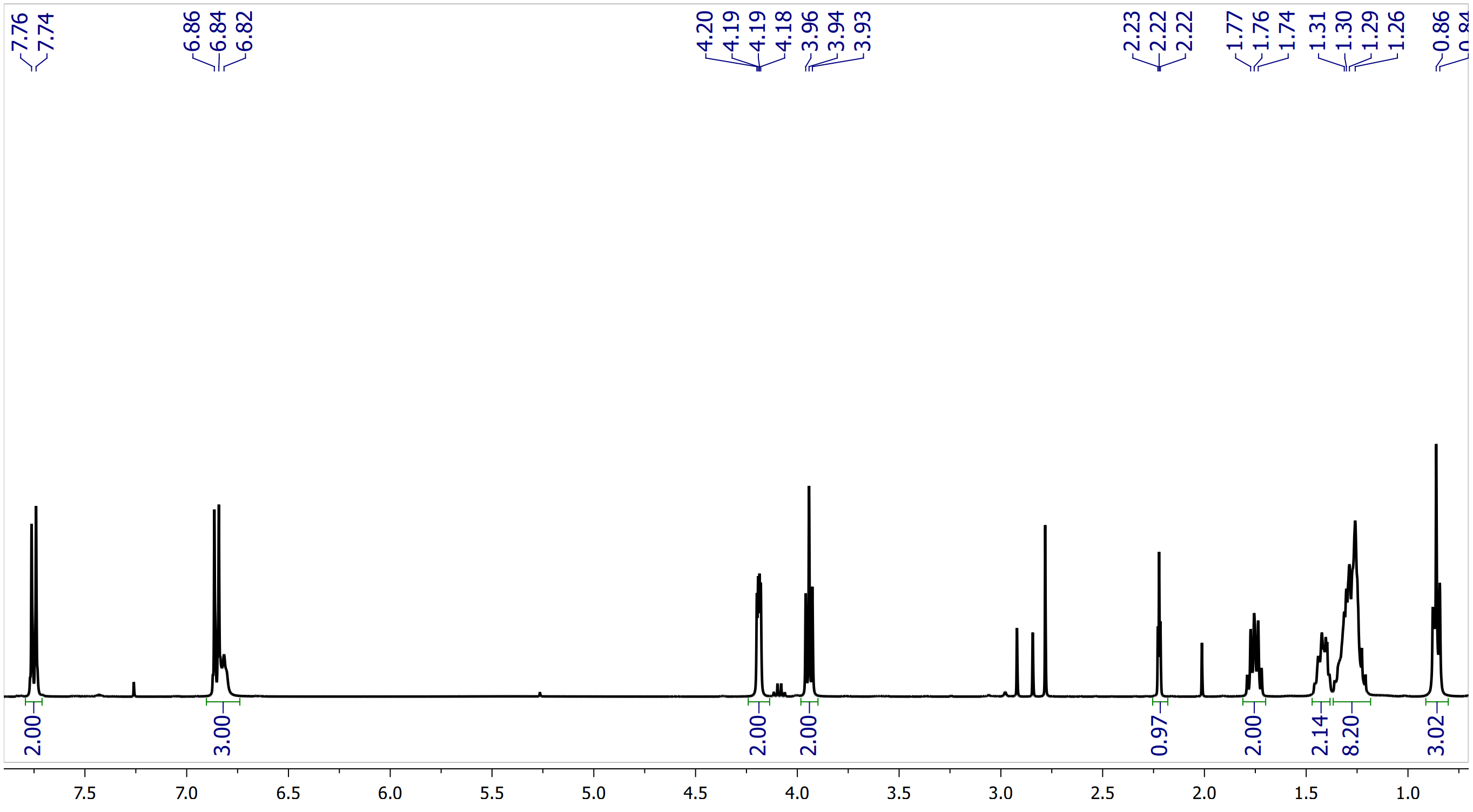


**Supplemental Figure S17**. ^1^H NMR of compound **11** in CDCl_3_.


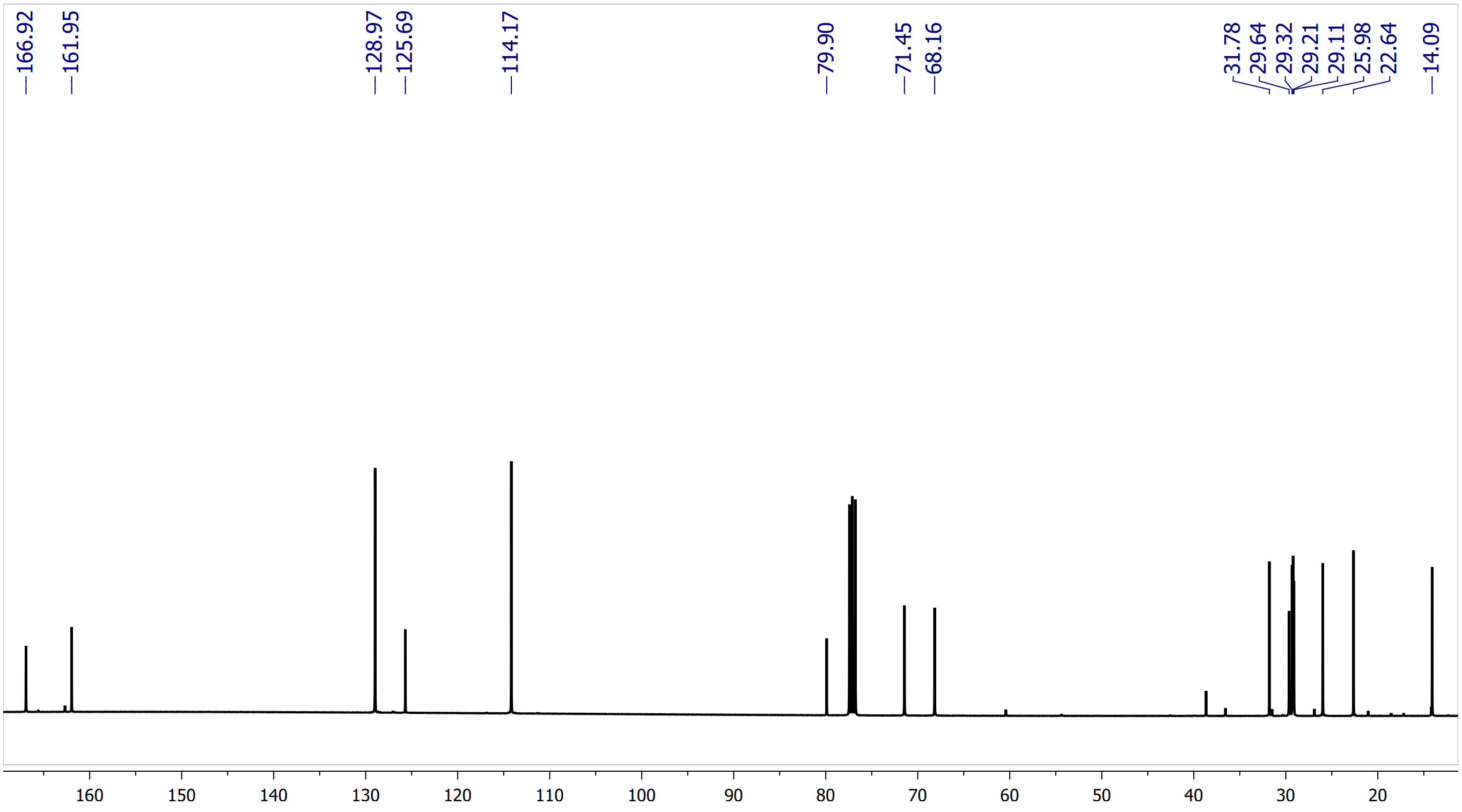


**Supplemental Figure S18**. ^13^C NMR of compound **11** in CDCl_3_.


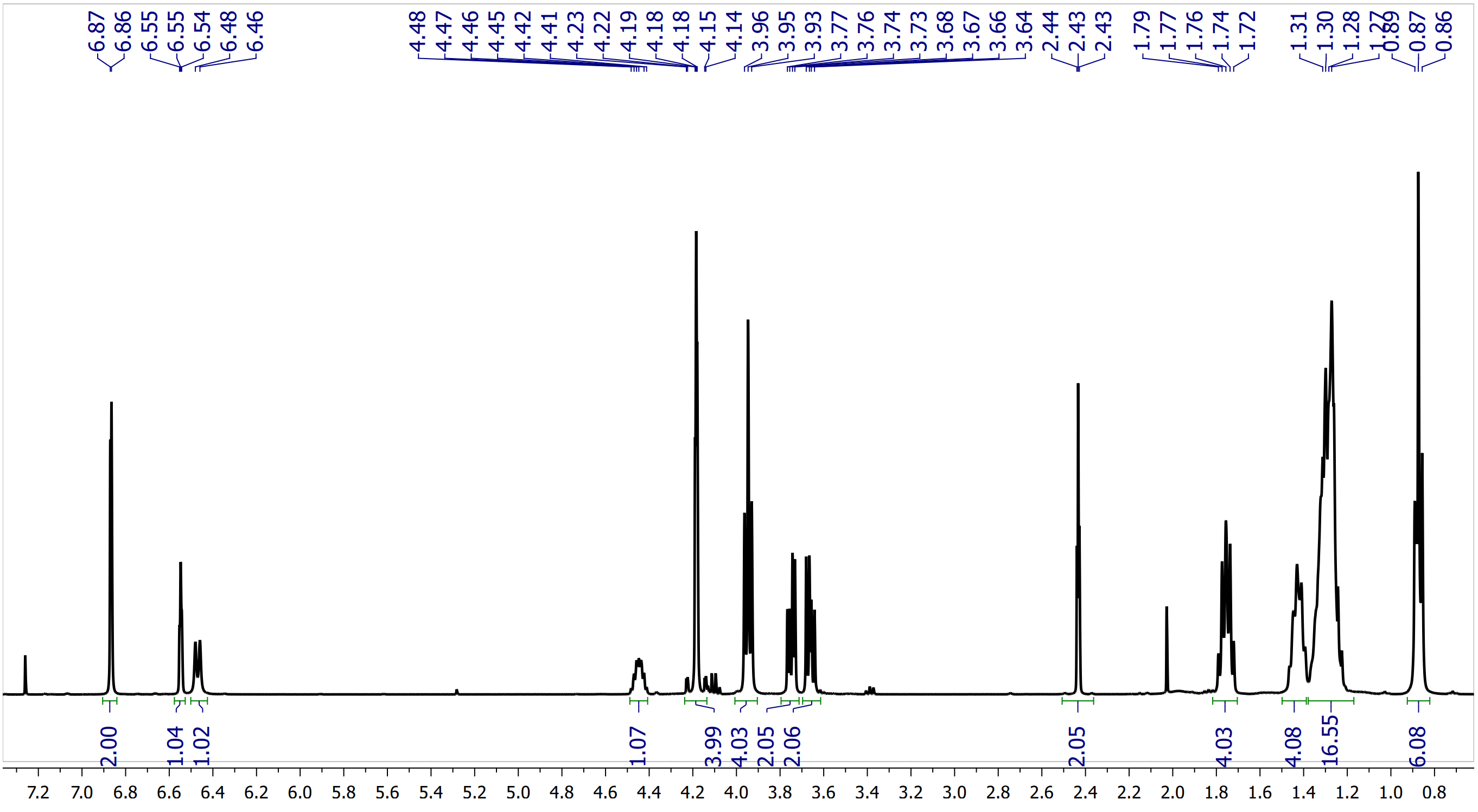


**Supplemental Figure S19**. ^1^H NMR of compound **17** in CDCl_3_.


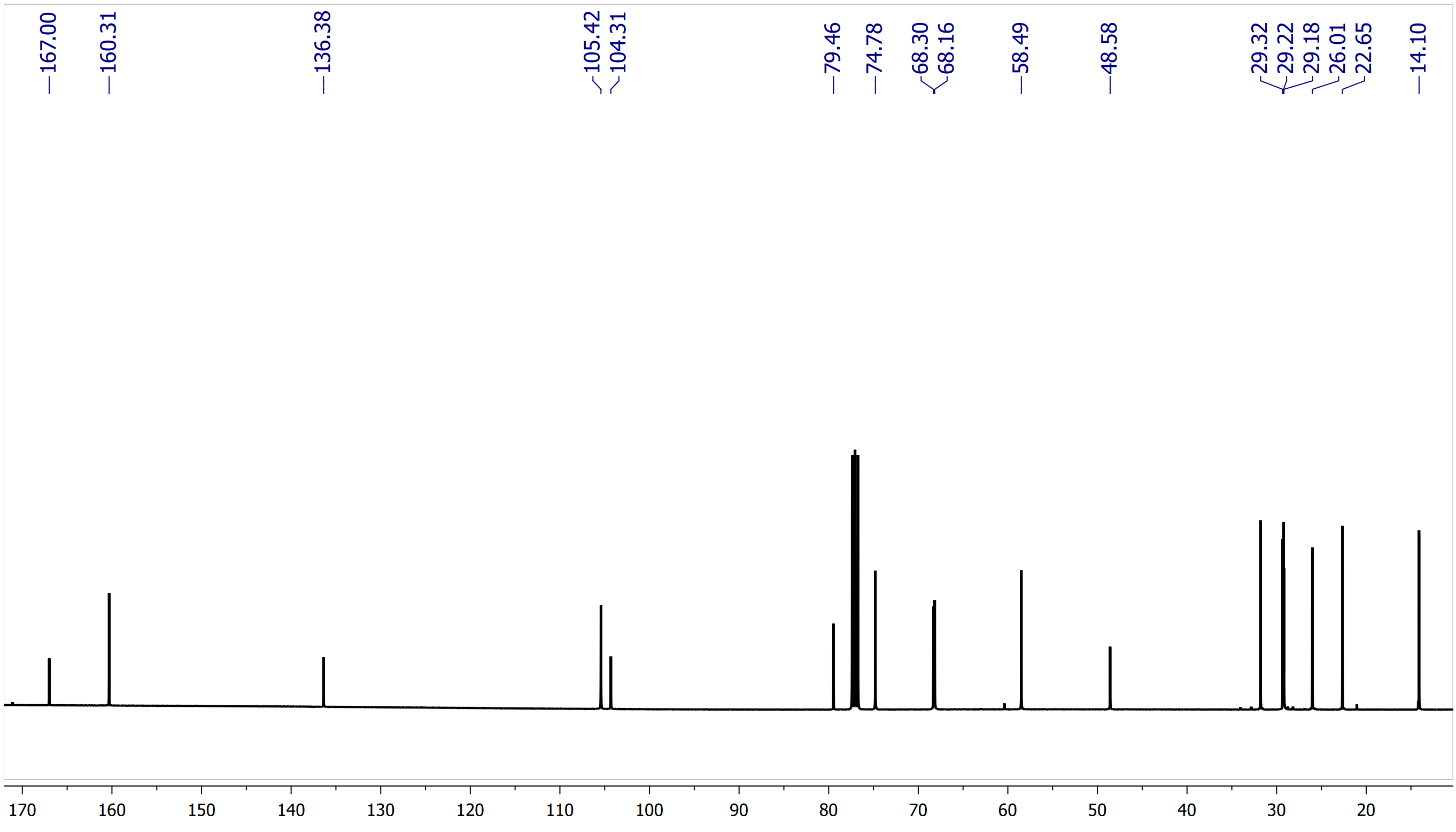


**Supplemental Figure S20**. ^13^C NMR of compound **17** in CDCl_3_.


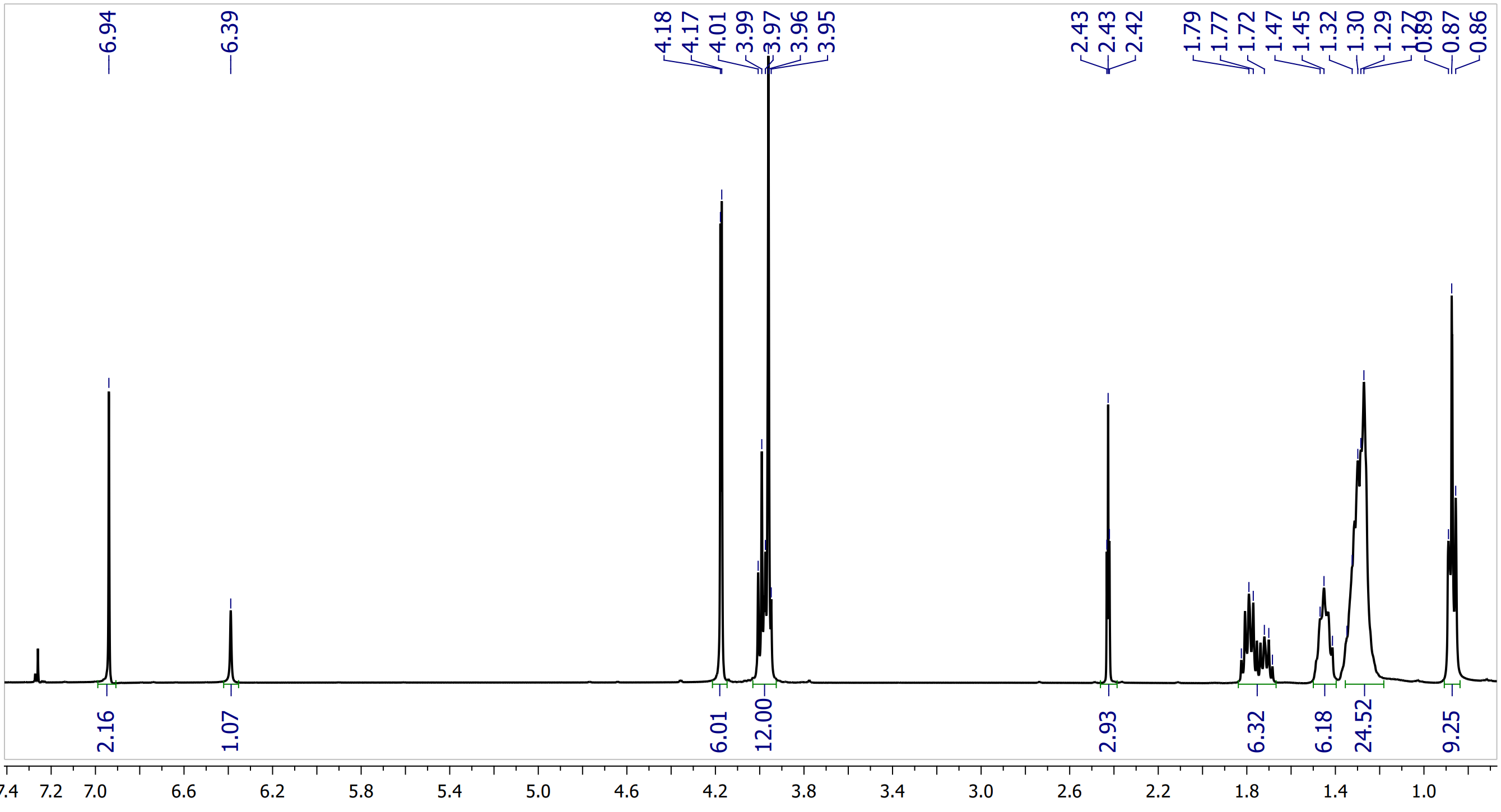


**Supplemental Figure S21**. ^1^H NMR of compound **23** in CDCl_3_.


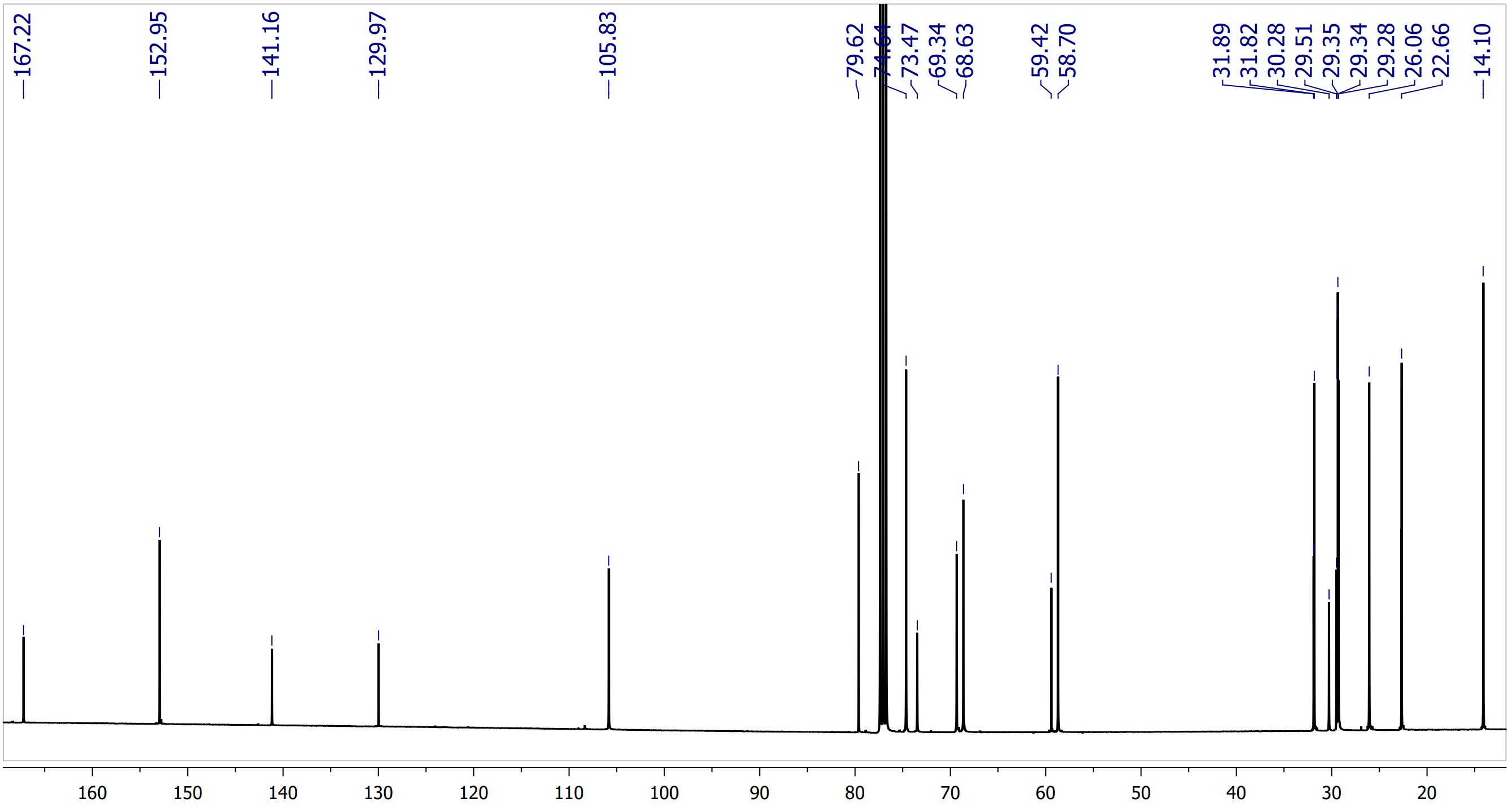


**Supplemental Figure S22**. ^13^C NMR of compound **23** in CDCl_3_.


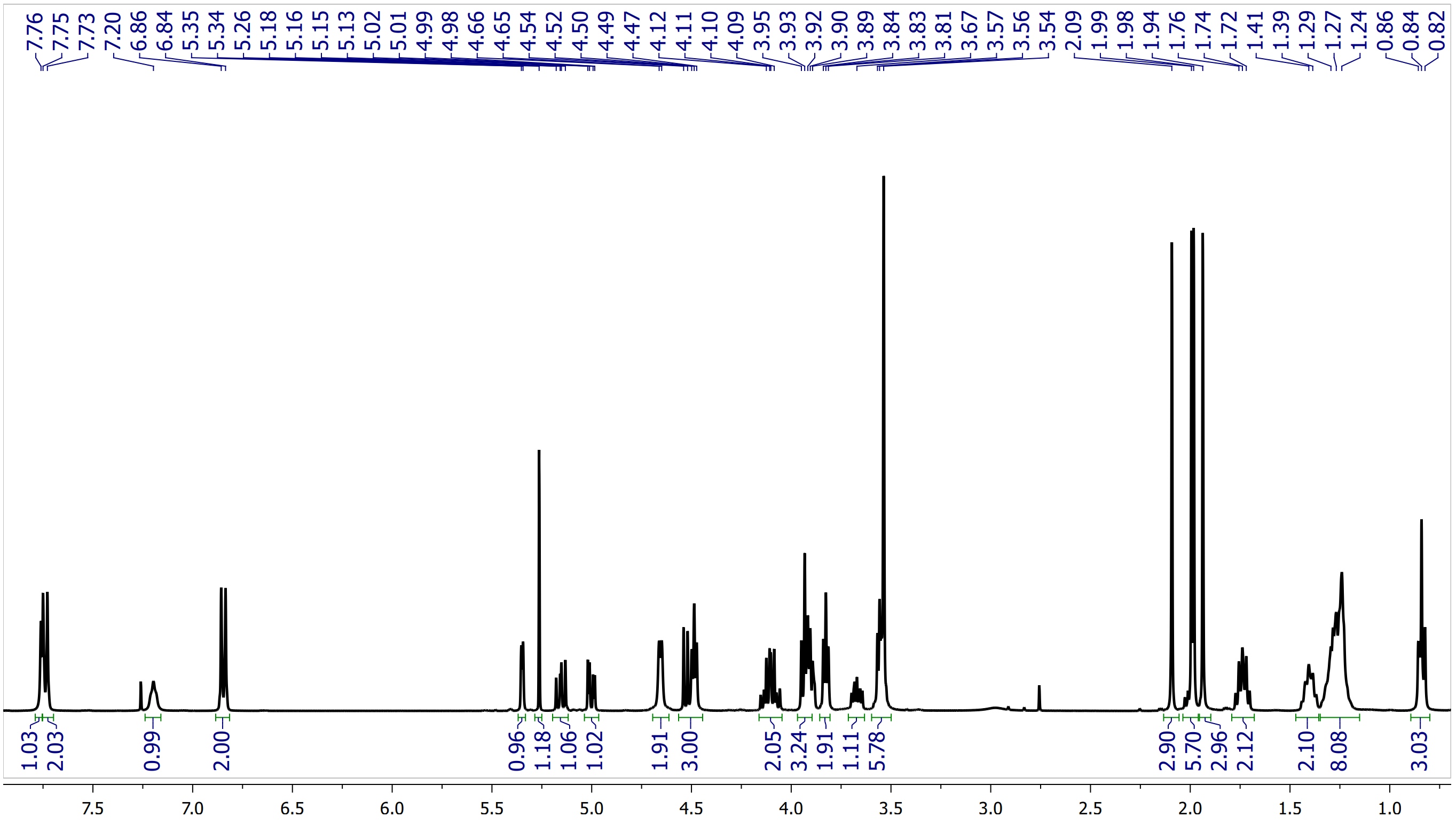


**Supplemental Figure S23**. ^1^H NMR of compound **12** in CDCl_3_.


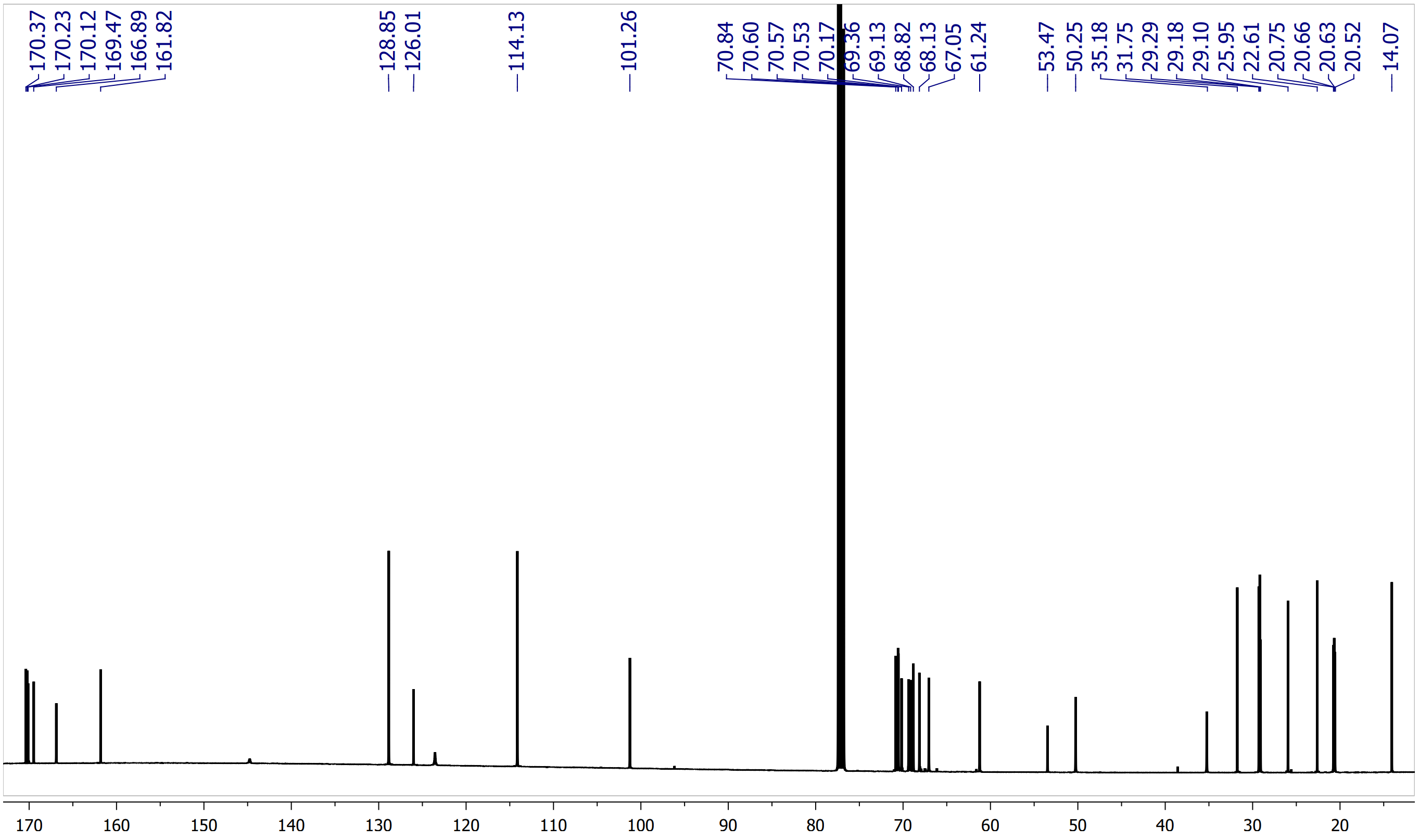


**Supplemental Figure S24**. ^13^C NMR of compound **12** in CDCl_3_.


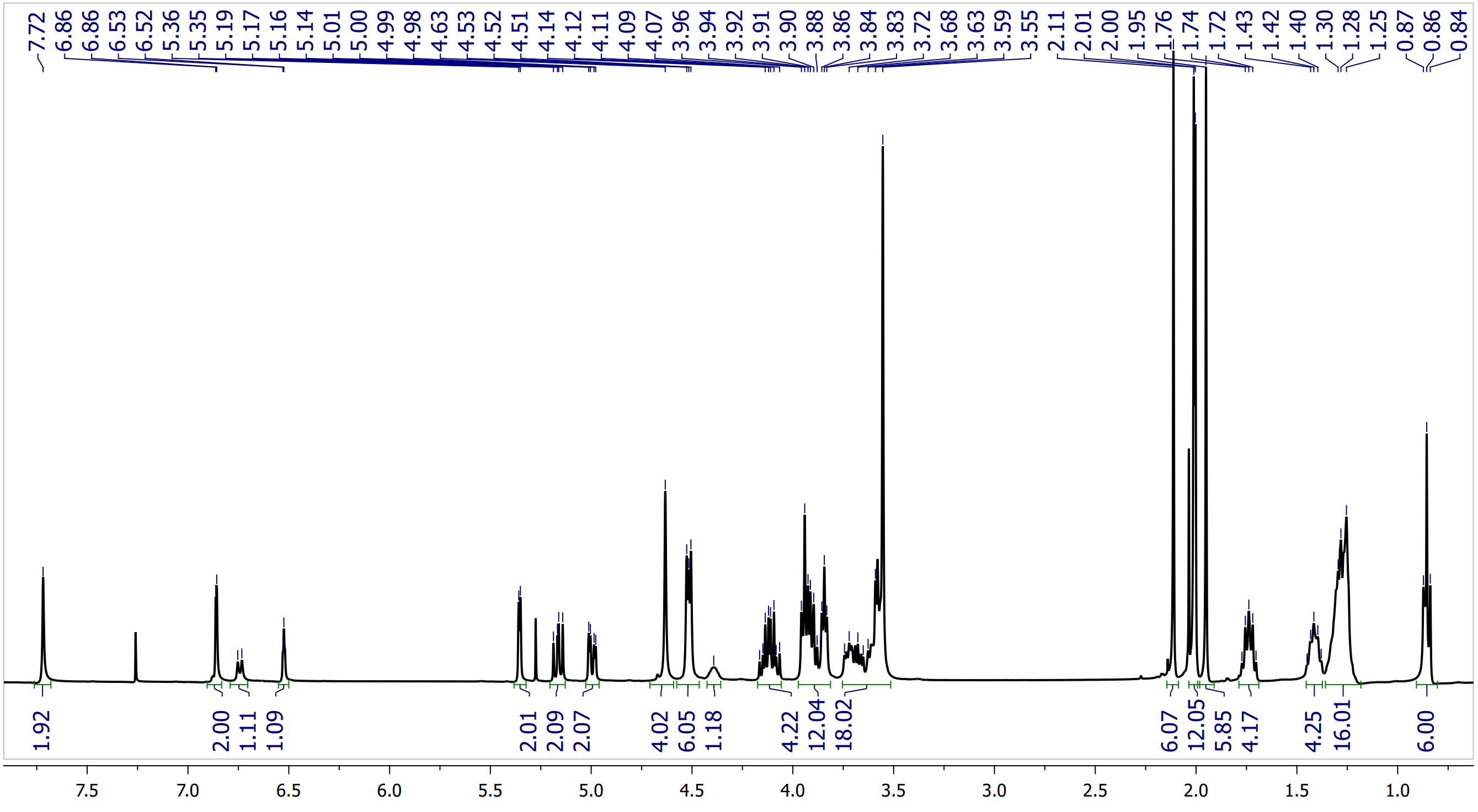


**Supplemental Figure S25**. ^1^H NMR of compound **18** in CDCl_3_.


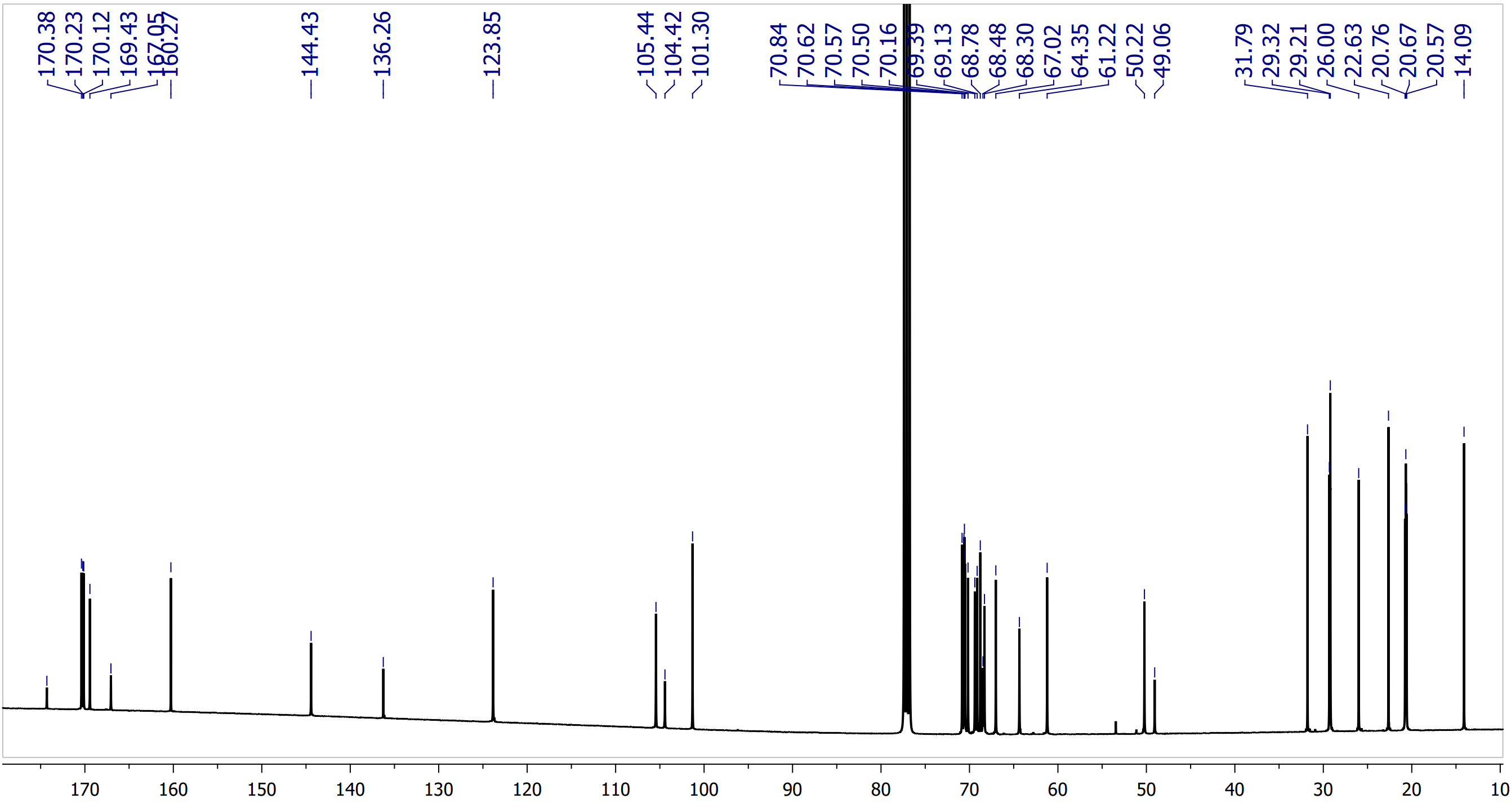


**Supplemental Figure S26**. ^13^C NMR of compound **18** in CDCl_3_.


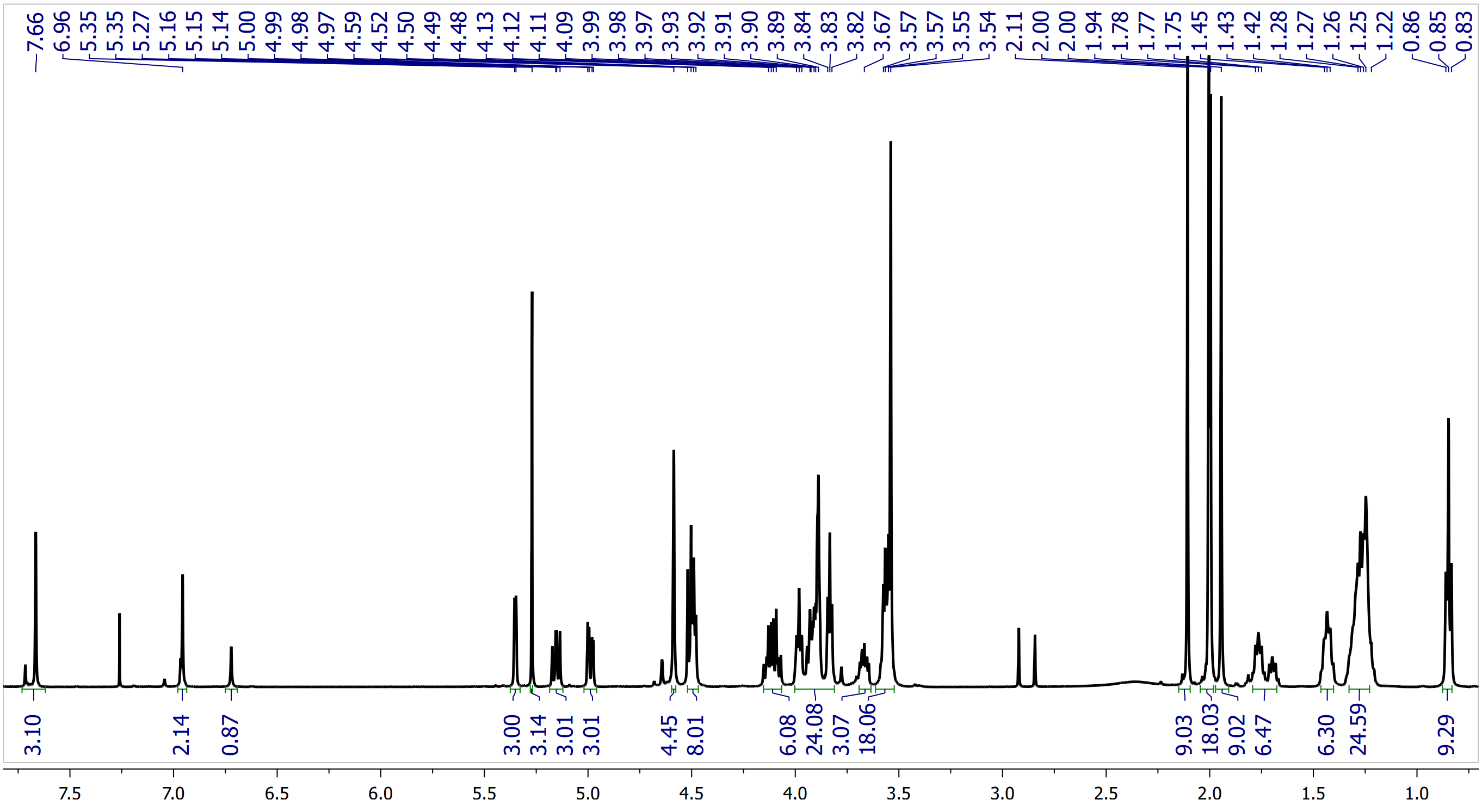


**Supplemental Figure S27**. ^1^H NMR of compound **24** in CDCl_3_.


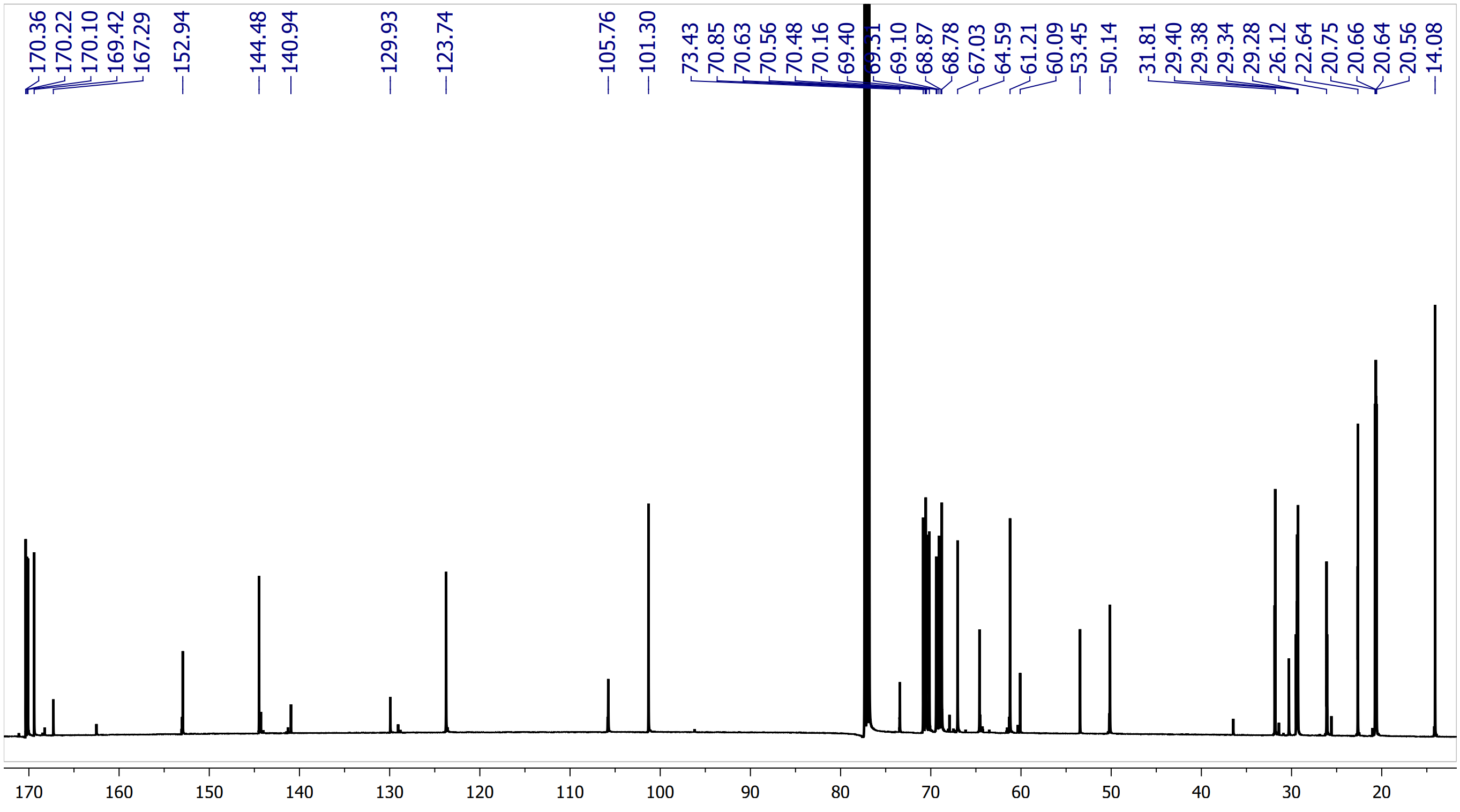


**Supplemental Figure S28**. ^13^C NMR of compound **24** in CDCl_3_.


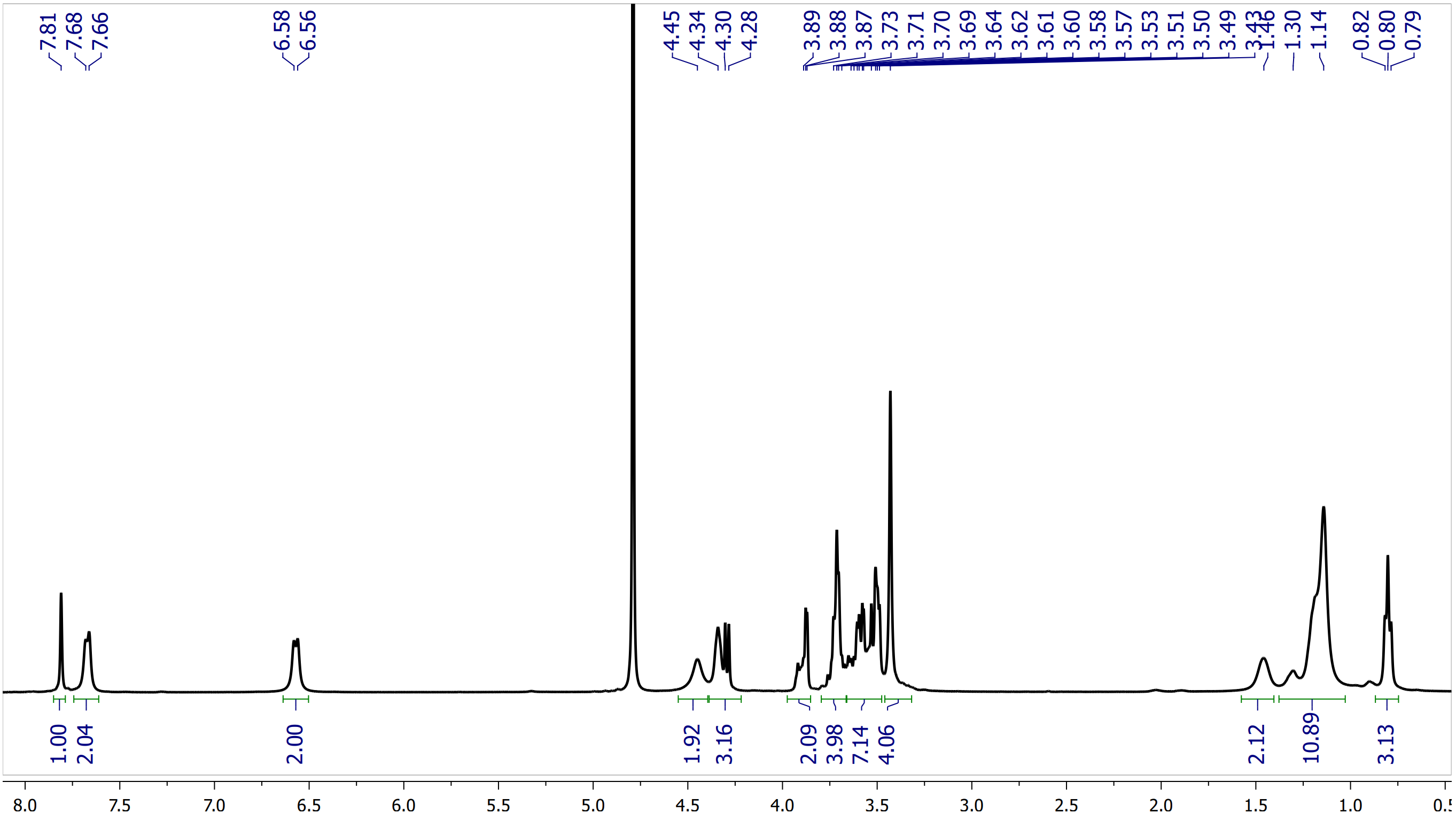


**Supplemental Figure S29**. ^1^H NMR of **Linear Amphiphile** in D_2_O.


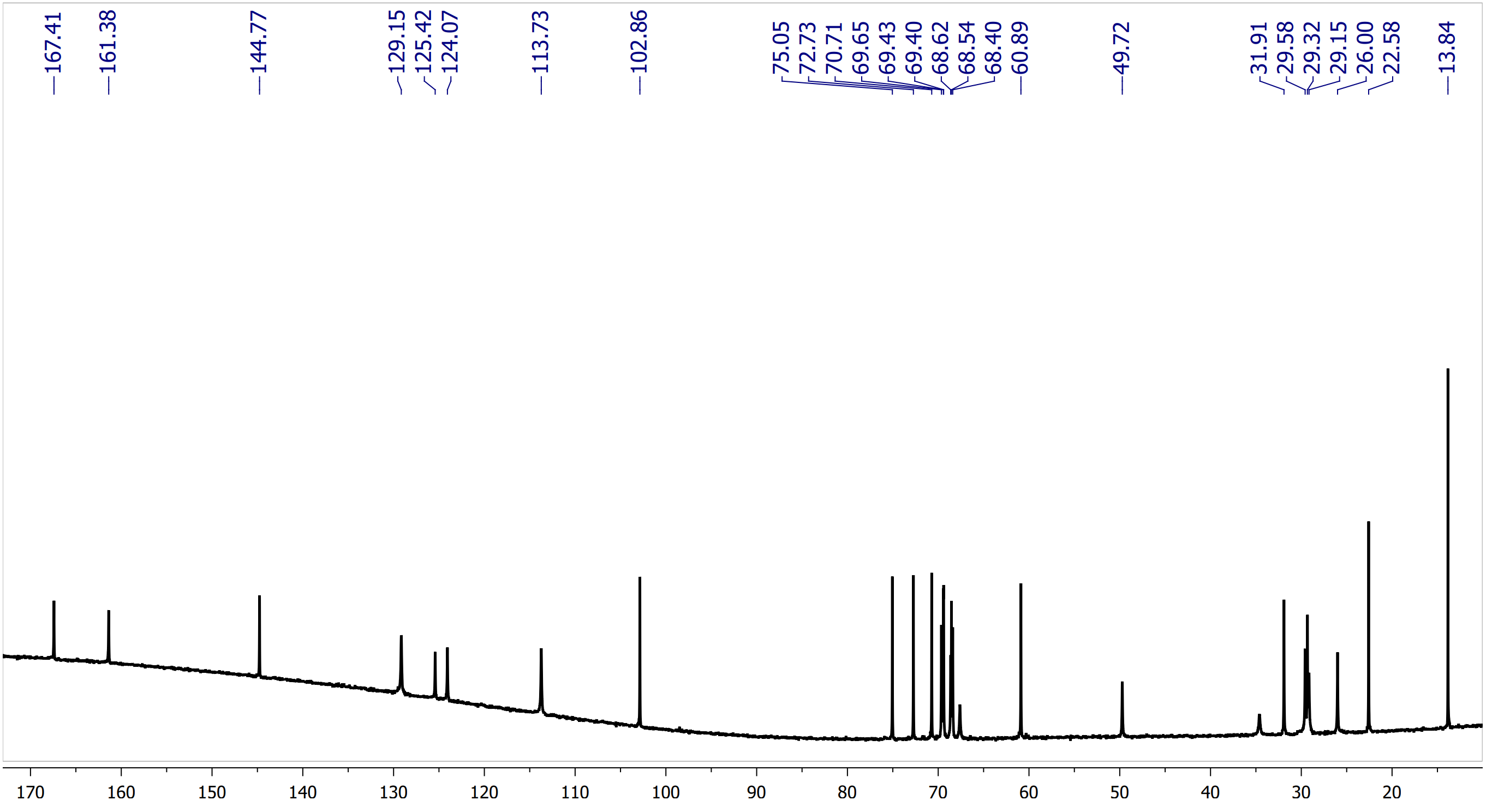


**Supplemental Figure S30**. ^13^C NMR of **Linear Amphiphile** in D_2_O.


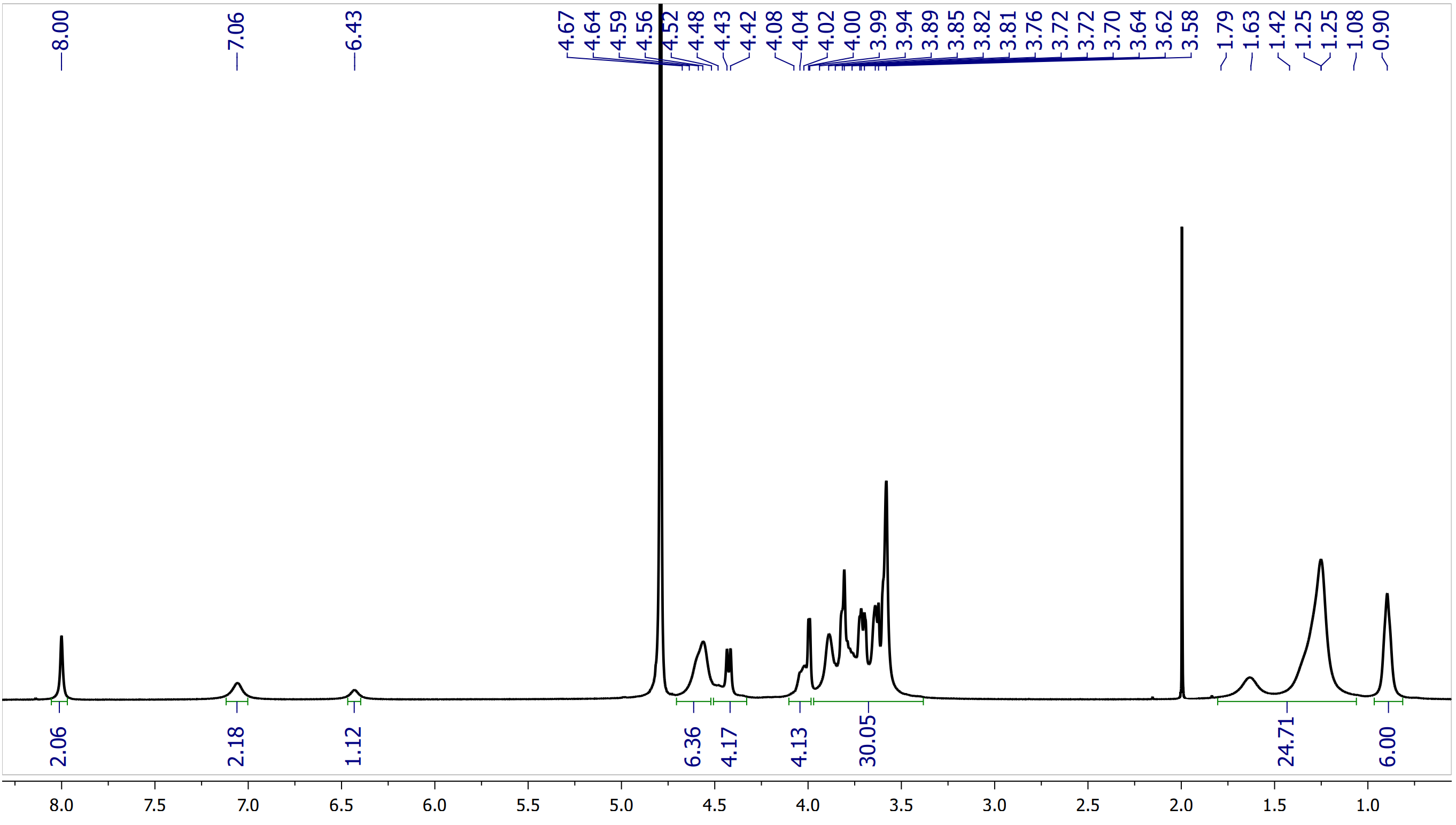


**Supplemental Figure S31**. ^1^H NMR of **Twinned Amphiphile** in D_2_O.


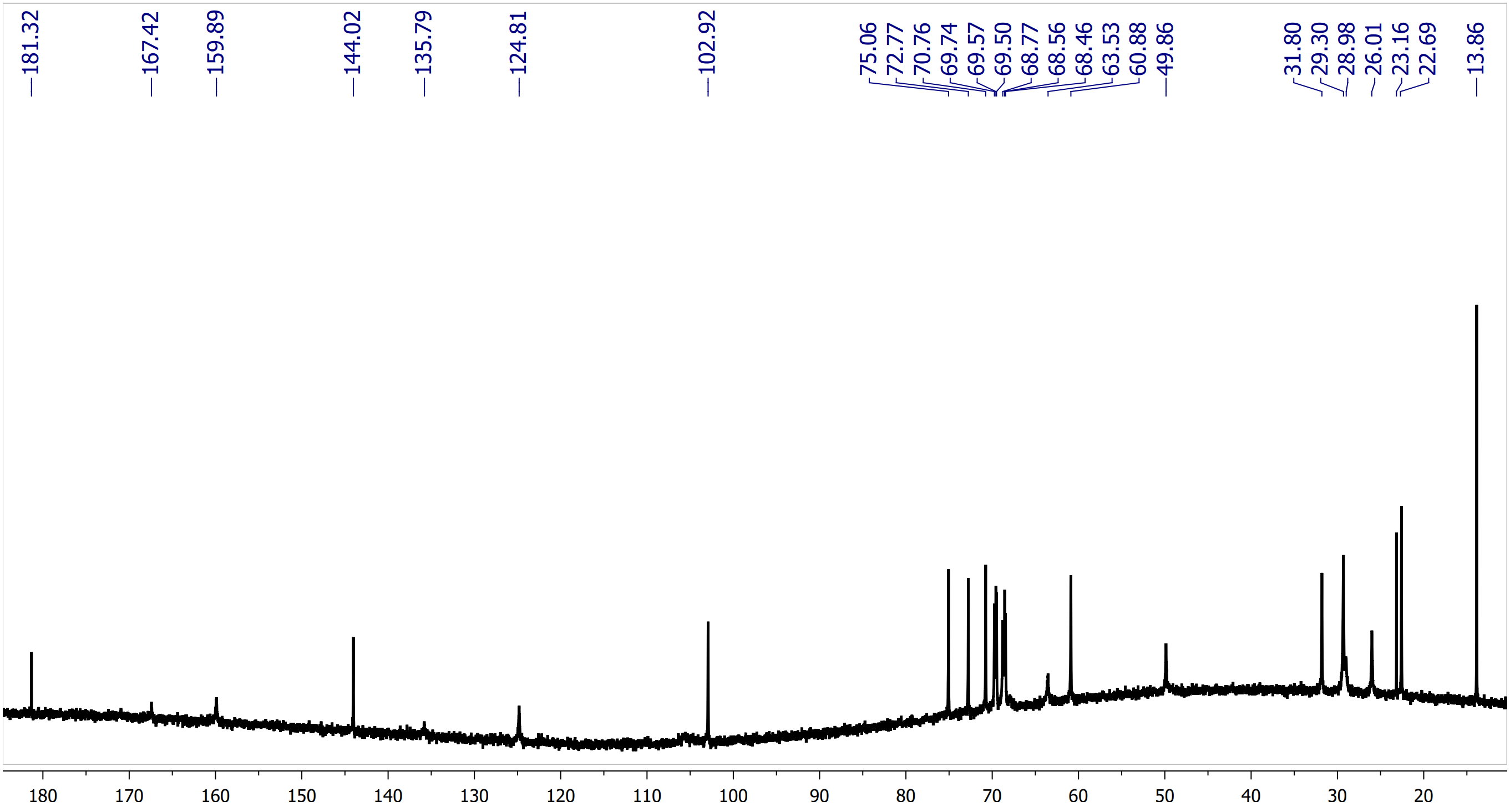


**Supplemental Figure S32**. ^13^C NMR of **Twinned Amphiphile** in D_2_O.


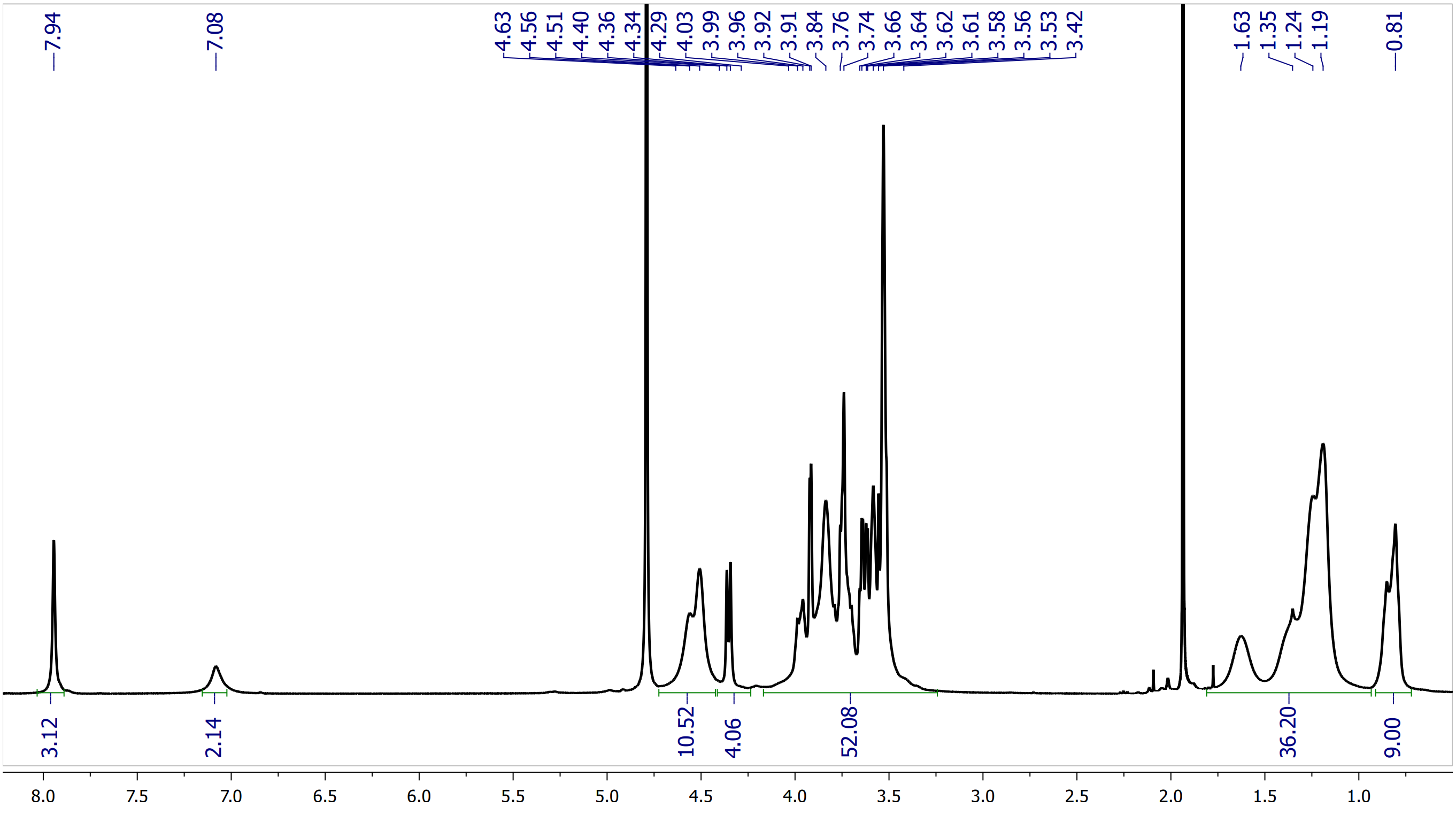


**Supplemental Figure S33.** ^1^H NMR of **Branched Amphiphile** in D_2_O.


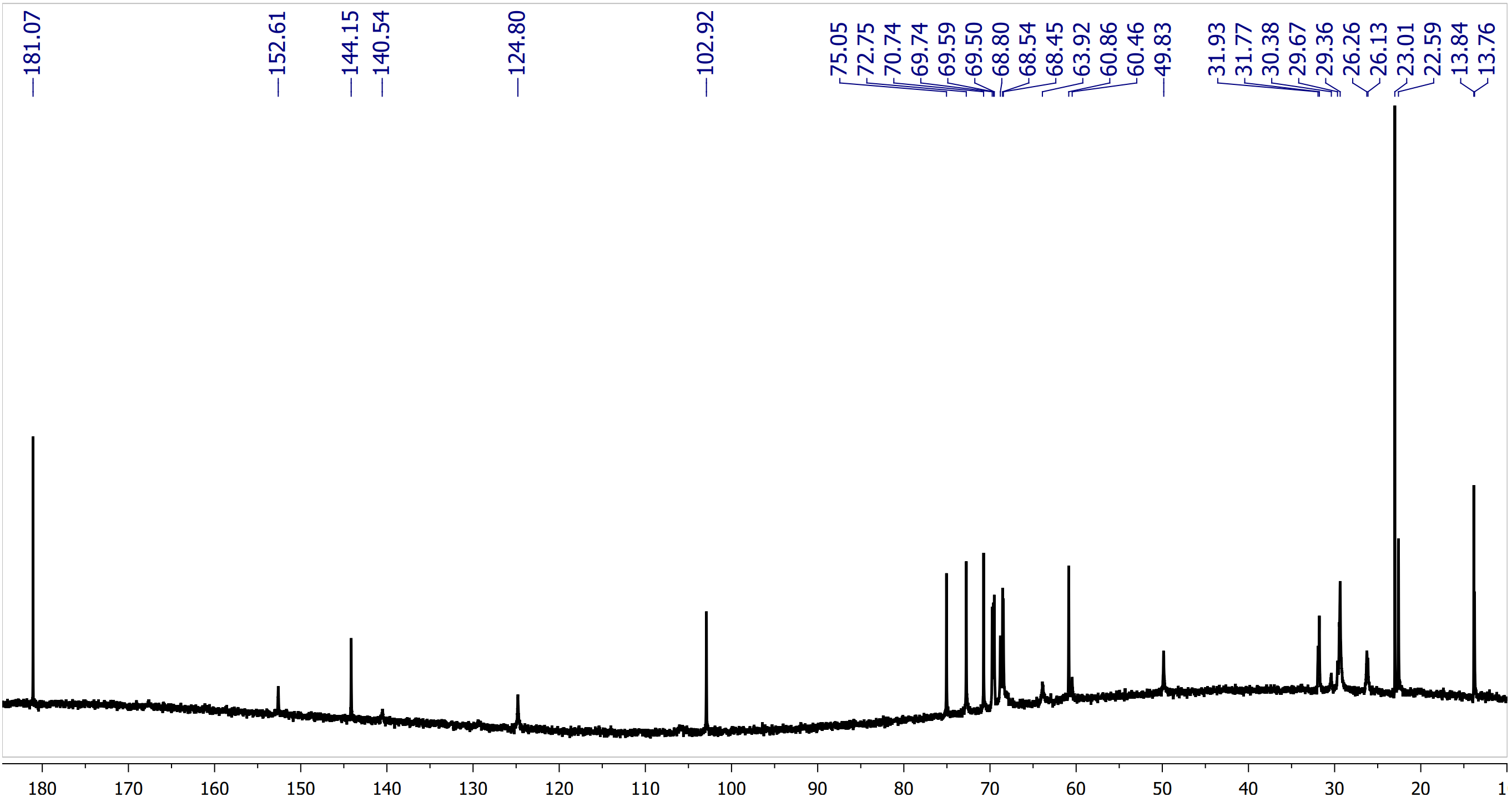


**Supplemental Figure S34**. ^13^C NMR of **Branched Amphiphile** in D_2_O.

**Supplemental Table S1**. Self-assembly behaviour of amphiphiles measured.

| **Amphiphile** | ^a^CMC (M) | DLS | cTEM |
| --- | --- | --- | --- |
|  |  | Size (d.nm) | Size (d.nm) |
| Linear | 1.94 × 10^-4^ | 8.64 ± 2.2 | 6.04 ± 1.4 |
| Twinned | 1.01 × 10^-4^ | 10.59 ± 2.9 | 8.48 ± 3.2 |
| Branched | 3.24 × 10^-5^ | 9.01 ± 2.4 | 7.22 ± 1.5 |

^a^Calculated using fluorescence measurement. ^b^DLS size distribution by Intensity.


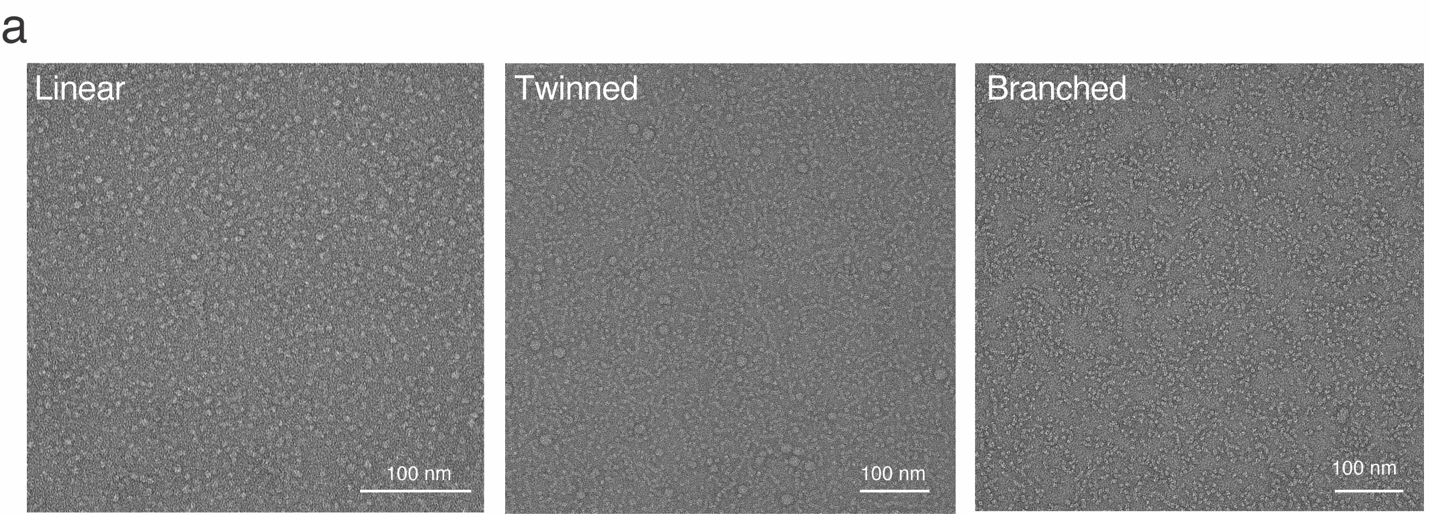


**Supplemental Figure S35. TEM micrographs of micelles prepared using galactose-modified amphiphiles**. a) TEM micrographs of linear, twinned and branched micelles with spherical structures obtained for linear (d=6.04 ± 1.4 nm) and branched (d=7.22 ± 1.5 nm), and worm-like and spherical structures obtained for twinned amphiphile (d=8.49 ± 3.2 nm).

*

*
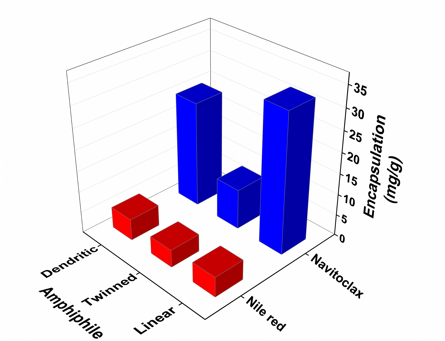

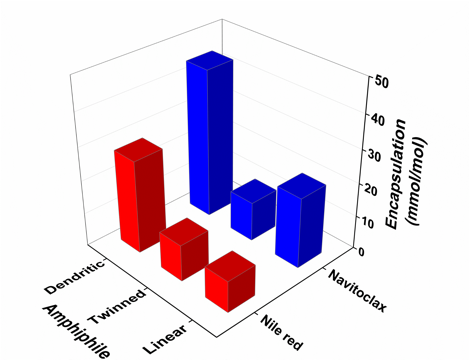


**Branched**

**Branched**

c

b

a

**Supplemental Figure S36.** Loading amounts of different amphiphiles with both Nile Red and Navitoclax (a) UV-vis spectra of encapsulated Nile red in all the three amphiphiles in water; (b) Nile red and Navitoclax encapsulation efficiency (mg/g) of amphiphiles; (c) Nile red and Navitoclax encapsulation efficiency (mmol/mol) of amphiphiles.

**Supplemental Table S2**. Self-assembly and drug encapsulation behaviour of amphiphiles measured.

| **Amphiphile** | ^c^HLB =  20 x M_h_/M_w_ | ^d^Nile red | | ^e^Navitoclax | |
| --- | --- | --- | --- | --- | --- |
|  |  | mmol/mol | mg/g | mmol/mol | mg/g |
| Linear | 13.41 | 9.42 | 4.8 | 21.16 | 33.1 |
| Twinned | 14.43 | 10.97 | 4.2 | 11.84 | 9.6 |
| Branched | 14.67 | 27.81 | 5.1 | 44.53 | 25.1 |

^a^Calculated using Griffin method, where M_h_ and M_w_ are molecular weights of hydrophilic part and full amphiphile, respectively. ^b^Encapsulation efficiency of amphiphiles for Nile red is calculated by Beer-Lambert’s law. ^c^Encapsulation efficiency of Navitoclax is measured through the HPLC measurement.


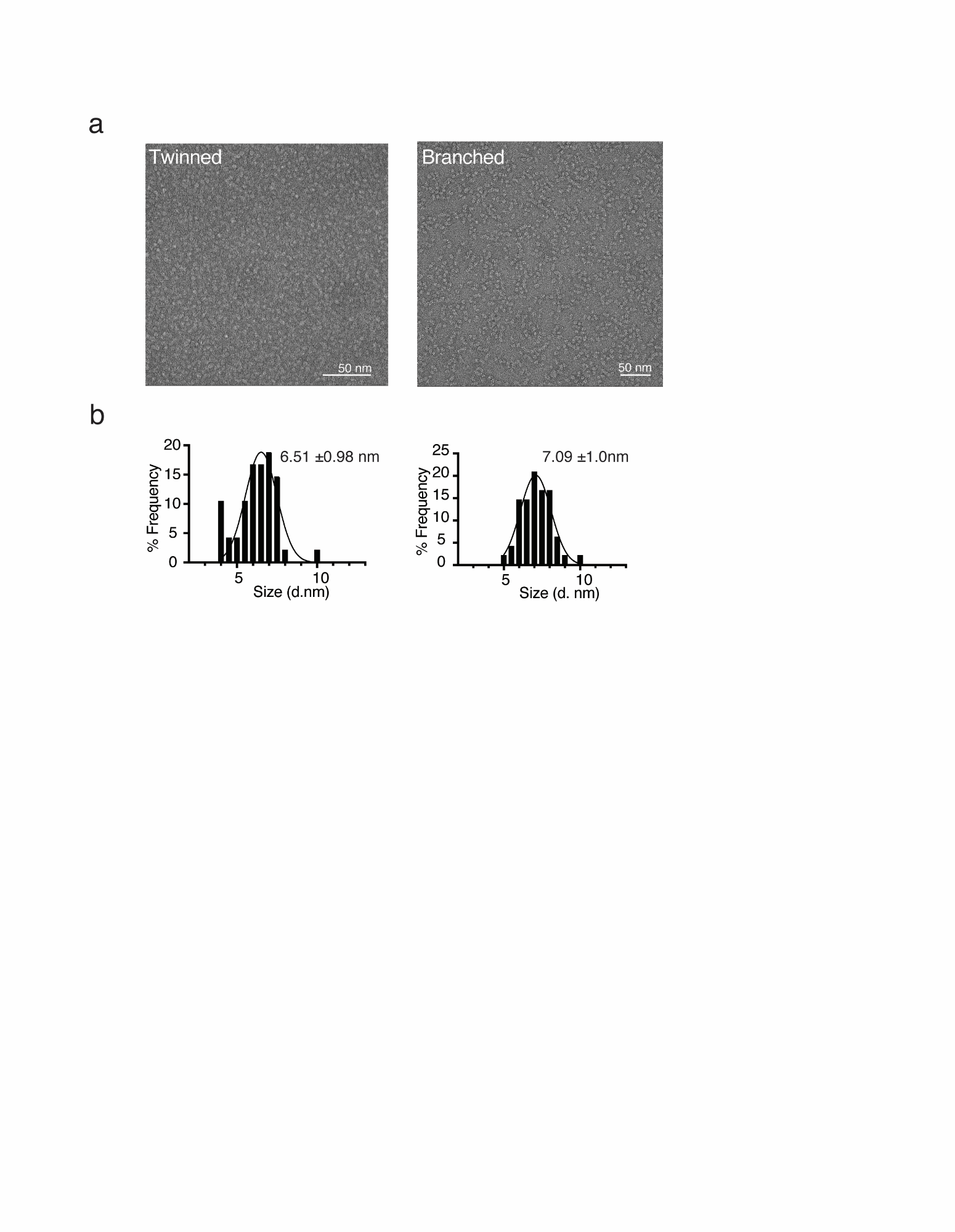


**Supplemental Figure S37. TEM micrographs of micelles using galactose-modified amphiphiles loaded with Nile Red**. a) TEM micrographs twinned and branched micelles with spherical structures obtained for branched (d=6.51 ± 0.98 nm) and for twinned amphiphile (d=7.09 ± 1.0 nm).


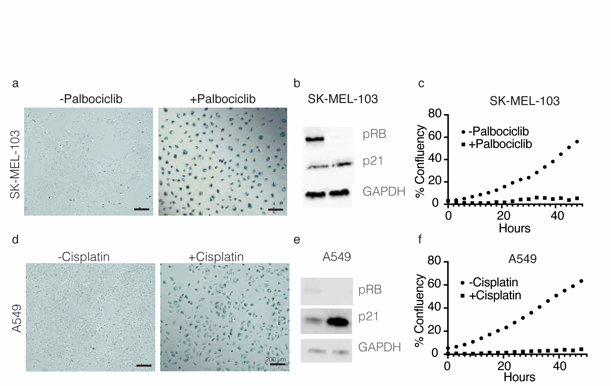


**Supplemental Figure S38. Characterization of Senescent Cells used in this study.** Cellular Senescence is defined as a cell with an increased expression of β-galactosidase (**a** and **d**), reduce growth capacity, (**b** and **e**) increased p21 expression and reduced expression of pRB. Growth curves (**c** and **f**) of control (black circles) and senescent (black squares) cells.


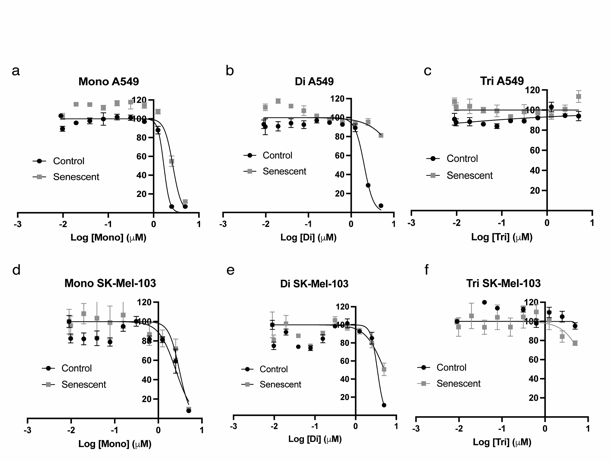


**Supplemental Figure S39. Toxicity of each amphiphile on the two cell lines both senescent and non senescent cells.** Viability curve of empty micelle of A549 cells a) Mono b) Di and c) Tri. Viability curves of empty micelles on SK-MEL-103 cells d) Mono e) Di and f) Tri. Senescence of A549 cells was induced by 15 µM Cisplatin (10 days). Senescence of SK-MEL-103 was induced by 5 µM Palbociclib (7 days). Data represent mean ± SD. Cell viability was assessed using the CellTiter-Blue assay.


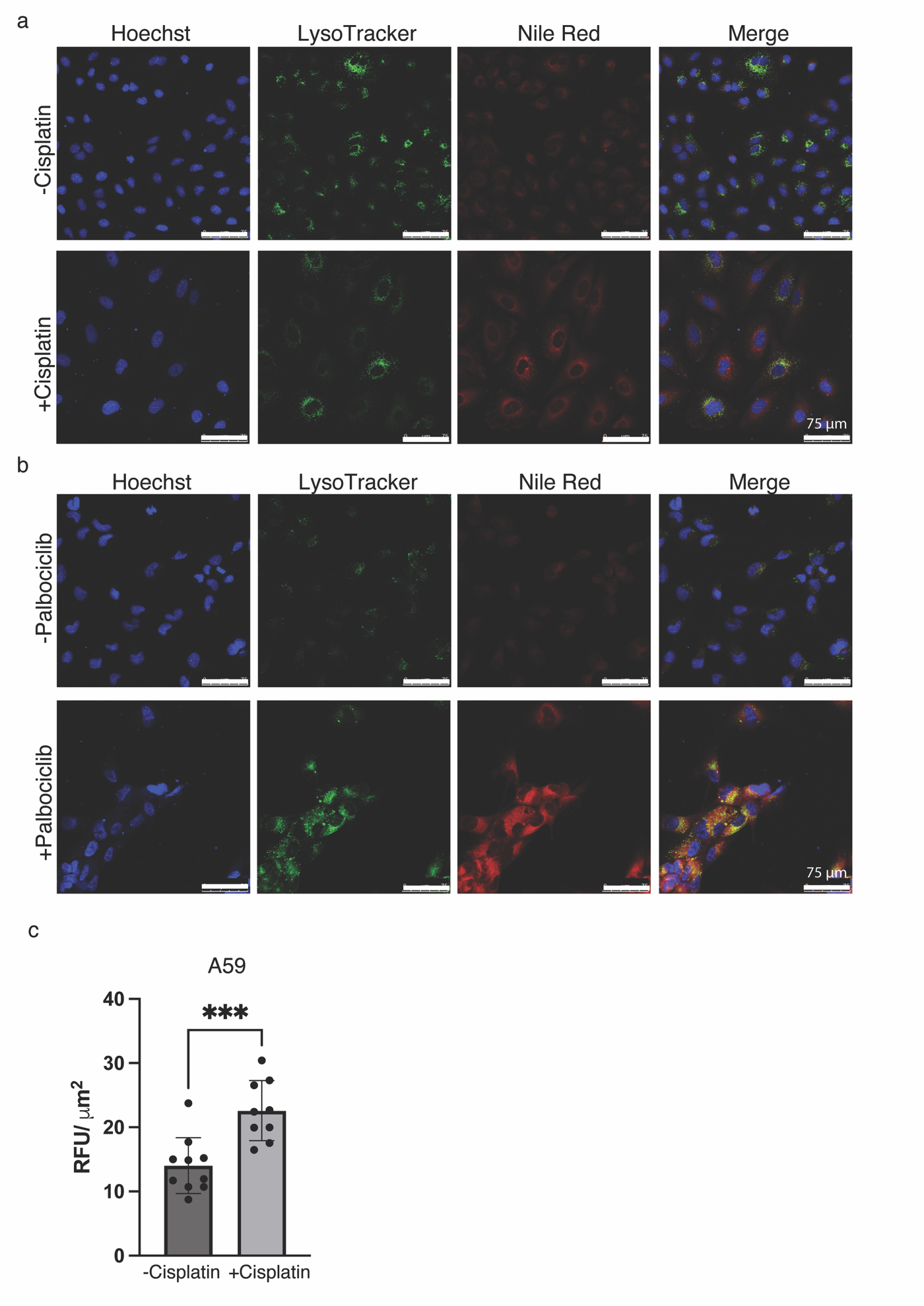


**Supplemental Figure S40. Demonstration of Lysosomal delivery of the micelles in both A549 and SK-MEL-103 cell lines. a**) Control and senescent (+Cisplatin) A549 cells incubated with trimeric micelle encapsulating nile red. **b)** Control and senescent (+palbociclib) SK-MEL-103 cells incubated with trimeric micelle encapsulating nile red. **c**) Quantification of a549 cells incubated with trimeric micelle encapsulating nile red. Nucleus was stained using Hoechst stain, lysosome with LysoTracker Green. Confocal images were obtained on a Leica SP5 confocal microscope. Data is obtained using confocal images, and represent mean ± SD, and a Two tailed t test was used to calculate the significance (*p < 0.05, **p <0.01, ***p < 0.001, and ****p<0.0001).

**
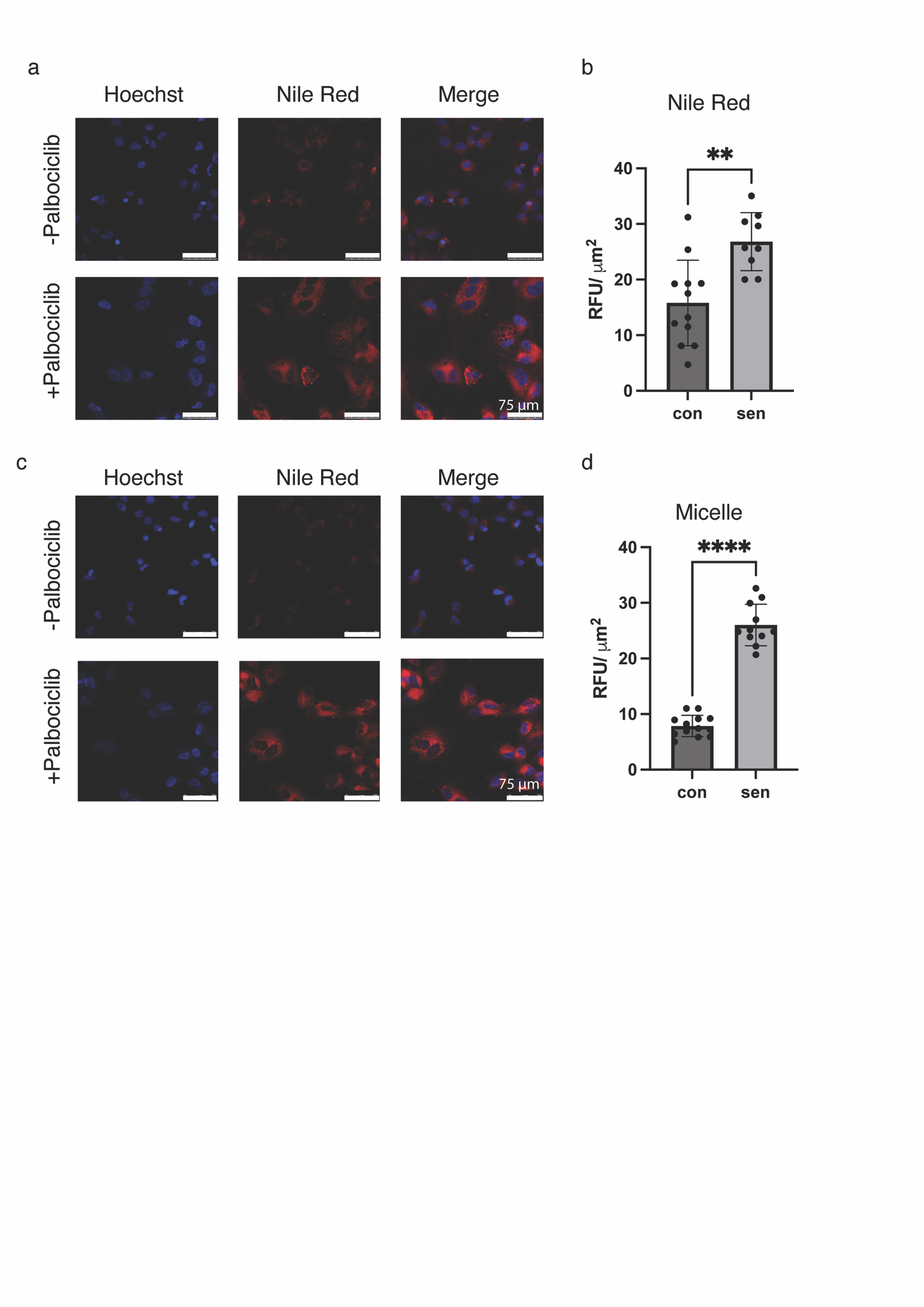
**

**Supplemental Figure S41. Comparison of nile red single molecule and nile red delivered by micelle. a**) Control and senescent (+Palbociclib) SK-MEL-103 cells incubated with nile red. **b**) Quantification of cells labeled with free nile red. **c)** Control and senescent (+palbociclib) SK-MEL-103 cells incubated with trimeric micelle encapsulating nile red.. Nucleus was stained using Hoechst stain, lysosome with LysoTracker Green. Confocal images were obtained on a Leica SP5 confocal microscope. Data is obtained using confocal images, and represent mean ± SD, and a Two tailed t test was used to calculate the significance (*p < 0.05, **p < 0.01, ***p < .001, and ****p<0.0001).
